# The Protective Effects of Live and Pasteurized Akkermansia muciniphila and Its Extracellular Vesicles against HFD/CCl4-Induced Liver Injury

**DOI:** 10.1128/Spectrum.00484-21

**Published:** 2021-09-22

**Authors:** Shahrbanoo Keshavarz Azizi Raftar, Fatemeh Ashrafian, Abbas Yadegar, Arezou Lari, Hamid Reza Moradi, Arefeh Shahriary, Masoumeh Azimirad, Helia Alavifard, Zhaleh Mohsenifar, Mehdi Davari, Farzam Vaziri, Arfa Moshiri, Seyed Davar Siadat, Mohammad Reza Zali

**Affiliations:** a Microbiology Research Center, Pasteur Institute of Iran, Tehran, Iran; b Mycobacteriology and Pulmonary Research Department, Pasteur Institute of Iran, Tehran, Iran; c Foodborne and Waterborne Diseases Research Center, Research Institute for Gastroenterology and Liver Diseases, Shahid Beheshti University of Medical Sciences, Tehran, Iran; d Clinical Research Department, Pasteur Institute of Iran, Tehran, Iran; e Systems Biomedicine Unit, Pasteur Institute of Iran, Tehran, Iran; f Department of Basic Sciences, School of Veterinary Medicine, Shiraz University, Shiraz, Iran; g Basic and Molecular Epidemiology of Gastrointestinal Disorders Research Center, Research Institute for Gastroenterology and Liver Diseases, Shahid Beheshti University of Medical Sciences, Tehran, Iran; h Taleghani Hospital, Department of Pathology, School of Medicine, Shahid Beheshti University of Medical Sciences, Tehran, Iran; i Gastroenterology and Liver Diseases Research Center, Research Institute for Gastroenterology and Liver Diseases, Shahid Beheshti University of Medical Sciences, Tehran, Iran; j Experimental Therapy Unit, Laboratory of Oncology, Giannina Gaslini Children’s Hospital, Genoa, Italy; University of Nebraska-Lincoln

**Keywords:** *Akkermansia muciniphila*, liver fibrosis, extracellular vesicles, hepatic stellate cells, intestinal bacteria

## Abstract

Akkermansia muciniphila, as a member of the gut microbiota, has been proposed as a next-generation probiotic. Liver fibrosis is the main determinant of liver dysfunction and mortality in patients with chronic liver disease. In this study, we aimed to determine the beneficial effects of live and pasteurized A. muciniphila and its extracellular vesicles (EVs) on the prevention of liver fibrosis. The response of hepatic stellate cells (HSCs) to live and pasteurized *A. muciniphila* and its EVs was examined in quiescent, lipopolysaccharide (LPS)-activated LX-2 cells. Liver fibrosis was induced in 8-week-old C57BL/6 mice, using a high-fat diet (HFD) and carbon tetrachloride (CCl4) administration for 4 weeks. The mice were concomitantly treated via oral gavage with three forms of bacteria. The relative expression of different fibrosis and inflammatory markers was assessed in the tissues. Histological markers, serum biochemical parameters, and cytokine production were also analyzed, and their correlations with the relative abundance of targeted fecal bacteria were examined. All *A. muciniphila* preparations exhibited protective effects against HSC activation; however, EVs showed the greatest activity in HSC regression. Oral gavage with *A. muciniphila* ameliorated the serum biochemical and inflammatory cytokines and improved liver and colon histopathological damages. The relative expression of fibrosis and inflammatory biomarkers was substantially attenuated in the tissues of all treated mice. The composition of targeted stool bacteria in the live *A. muciniphila* group was clearly different from that in the fibrosis group. This study indicated that *A. muciniphila* and its derivatives could successfully protect against HFD/CCl4-induced liver injury. However, further studies are needed to prove the beneficial effects of *A. muciniphila* on the liver.

**IMPORTANCE** Akkermansia muciniphila, as a member of the gut microbiota, has been proposed as a next-generation probiotic. Liver fibrosis is the main determinant of liver dysfunction and mortality in patients with chronic liver disease. In this study, we aimed to determine the beneficial effects of live and pasteurized *A. muciniphila* and its extracellular vesicles (EVs) on the prevention of liver fibrosis. The results of the present study indicated that oral administration of live and pasteurized *A. muciniphila* and its EVs could normalize the fecal targeted bacteria composition, improve the intestinal permeability, modulate inflammatory responses, and subsequently prevent liver injury in HFD/CCl4-administered mice. Following the improvement of intestinal and liver histopathology, HFD/CCl4-induced kidney damage and adipose tissue inflammation were also ameliorated by different *A. muciniphila* treatments.

## INTRODUCTION

Chronic liver disease (CLD) refers to liver dysfunction as a consequence of progressive destruction and regeneration of liver tissue, which can lead to fibrosis and hepatic carcinoma with high mortality rates (1). A group of liver diseases, including hepatitis B virus (HBV) infection, hepatitis C virus (HCV) infection, alcoholic liver disease, nonalcoholic fatty liver disease (NAFLD), and nonalcoholic steatohepatitis (NASH), have great impacts on the global burden of liver disease (2). Irrespective of the etiology, chronic hepatocyte injury and inflammatory response lead to scarring and subsequent fibrosis in the liver tissue (3). It is known that fibrosis is a wound-healing process that activates hepatic stellate cells (HSCs) following exposure to chronic stimuli and persistent inflammation; this results in the overexpression of the extracellular matrix (ECM) and a collagen-rich tissue that replaces the natural parenchyma of the liver (4).

Due to the increased development of new antiviral medications, as well as the increased prevalence of obesity and its related complications in recent decades, CLD is potentially becoming a nonviral disease worldwide (5). NAFLD, as the most prevalent chronic liver disease, is characterized by mild to severe steatosis, known as NASH, which is accompanied by fibrosis (6). CLD also affects other tissues through interstitial communication between the liver, gut, adipose, and kidney tissues (7, 8).

Akkermansia muciniphila, a bacterium that inhabits the gastrointestinal mucus layer, has been recently considered a next-generation probiotic strain in the treatment of obesity-related disorders (9). This mucinophilic bacterium contributes to the improvement of metabolic status by affecting the immune and metabolic pathways and increasing the intestinal barrier integrity. These positive changes occur through direct interactions of A. muciniphila-derived outer membrane proteins (OMPs), metabolites, and ligands with other members of the intestinal microbial community (10).

In recent years, particular attention has been paid to the use of nonviable bacterial supplementations as alternative products to reduce the potential risks of live bacteria, especially in high-risk individuals, such as immunocompromised patients (11). Administration of live or inactivated pasteurized *A. muciniphila* can greatly reduce the progression of metabolic disorder-associated diseases; also, the safety of *A. muciniphila* products has been recently reported in humans (12–14). We demonstrated that heat-killed *A. muciniphila* MucT strain was able to ameliorate LPS-induced HSC activation through modulation of fibrosis markers (15).

Bacterial extracellular vesicles (EVs) are submicron-sized bilayer lipid structures that are derived from the cell membrane of both Gram-negative and Gram-positive bacteria and can interact not only with the host cells but also with other microbiota (16, 17). The EVs of beneficial gastrointestinal bacteria play an immunomodulatory role through Toll-like receptor (TLR) signaling, especially TLR-2 and TLR-4 signaling (18). They also enhance the expression of tight-junction proteins, modulating the cytokine secretion and regulating the intestinal hemostasis (19).

Moreover, *A. muciniphila*-derived EVs seem to improve the gut barrier integrity in high-fat-diet (HFD)-induced diabetes by penetrating the large intestine and spreading to peripheral tissues, such as muscle, adipose, and liver tissues (20). Naturally, the gut microbiota is in complete synergy with the host and exerts remarkable effects, such as modulation of the immune system, prevention of pathogen colonization, and improvement of the digestion process and absorption of nutrients in the body (21). The liver and the gut have a bidirectional cross talk, and various liver disorders have been associated with an altered gut microbiome. The liver is the first organ to be exposed to a large number of intestinal microbial components and metabolites; therefore, maintaining a healthy intestinal environment can guarantee the liver health (22, 23).

In the present study, we used a meta-analysis of seven previously collected carbon tetrachloride (CCl4)-induced liver injury microarray data sets (24–30) to identify fibrosis-related genes. HSC responses to live and pasteurized *A. muciniphila* and its EVs were examined in quiescent LPS-activated LX-2 cell line to determine if EVs could reduce expression of fibrosis-related genes similar to what we had observed for heat-killed *A. muciniphila* (15). Next, the antifibrotic and anti-inflammatory effects of live and pasteurized *A. muciniphila* and its EVs were examined in the histopathology of liver and colon tissues of a mouse model of HFD/CCl4-induced liver injury. The gene expression of various inflammatory and fibrosis markers was evaluated in the mouse liver tissue. To determine if this protection against fibrosis occurred in other organs, as well, we also measured the expression of fibrosis and inflammatory marker genes in the epididymal white adipose tissue (eWAT), colon tissue, and kidney tissue. The fecal bacterial quantification was also performed in stool samples obtained at the end of study at different taxonomic levels, using real-time PCR method in all groups. Finally, the effects of different forms of *A. muciniphila* on the HSC activation, fibrotic and inflammatory markers, and fecal targeted bacteria alterations were compared *in vitro* and *in vivo*.

## RESULTS

### Fibrosis markers in mouse models of CCL4-induced liver injury.

**FIG 1:**
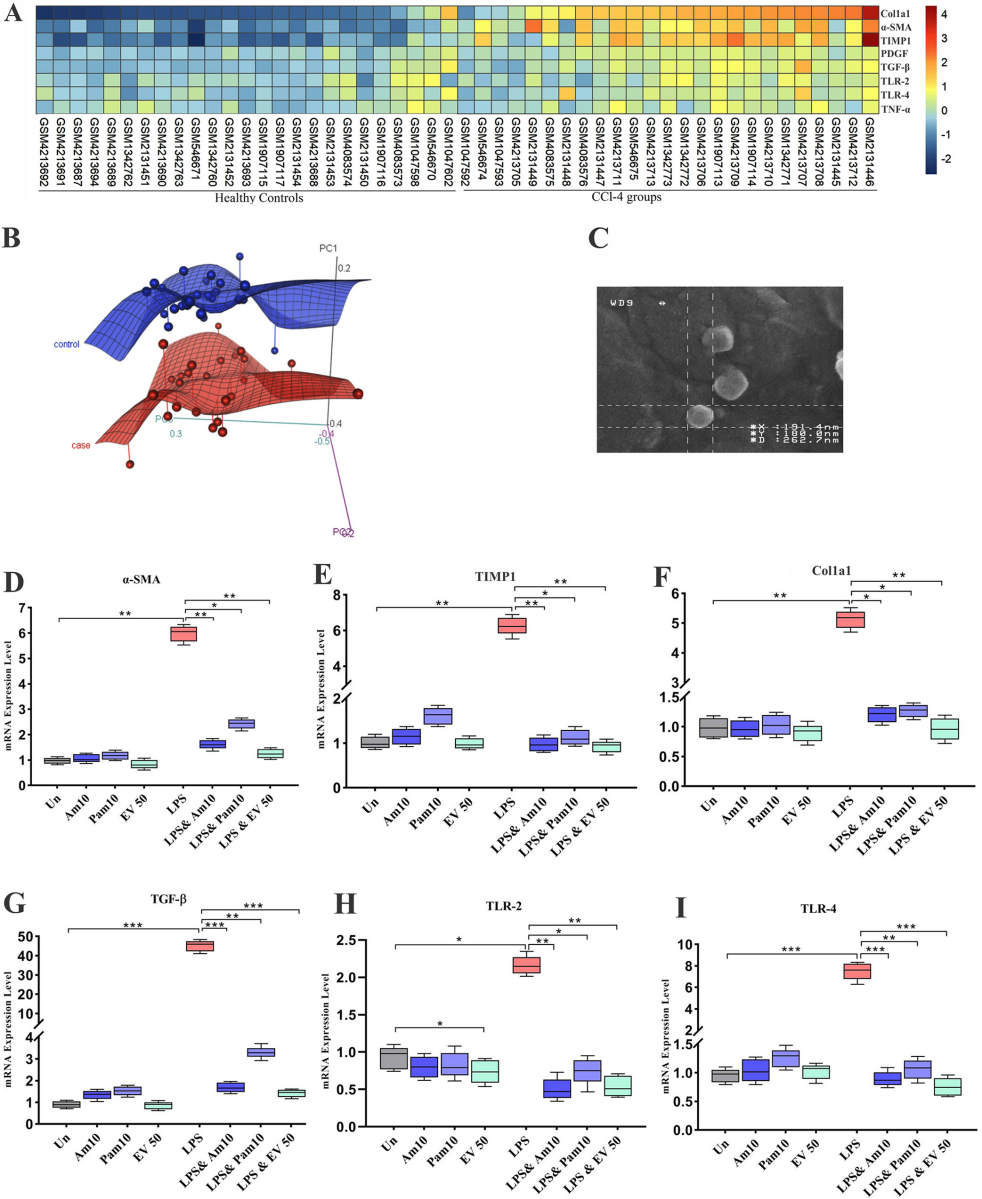
(A) A heatmap plot of microarray data sets meta-analysis. A *P* value of <0.05 was considered statistically significant by Mann-Whitney method. (B) PCA plot shows separated cluster between two groups. (C) Scanning electron microscopy shows spherical morphology of EVs (magnification: ×60,000). Inhibition of HSC activation and protective effects of all *A. muciniphila* supplementations in LX-2 cell line. mRNA level of HSCs activation-related genes, (D) α-SMA, (E) TIMP1, (F) Col1a1, (G) TGF-β, (H) TLR-2, and (I) TLR-4, in quiescence and LPS-activated LX-2 cells. Data are expressed as mean ± standard deviation (SD) (*n *= 5). *, *P < *0.05; **, *P < *0.01; and ***, *P < *0.001 by *post hoc* Turkey’s one-way ANOVA.

The meta-analysis performed on data sets comparing mRNA levels in mice with CCL4-induced liver injury to those in controls shows that the expression of eight genes, including tumor necrosis factor alpha (TNF-α), TLR-2, TLR-4, transforming growth factor beta (TGF-β), alpha smooth muscle actin (α-SMA), platelet-derived growth factor (PDGF), tissue inhibitor of metalloproteinases (TIMP1), and collagen type 1 alpha 1 (Col1a1) in liver, are closely related to fibrosis and were differentially expressed, with a *P* value of <0.05 by the Mann-Whitney method. As shown in the heatmap (Fig. 1A), inflammatory mediators were expressed significantly, with higher levels in the CCL4-induced liver fibrosis mice than in healthy controls (*P* value TLR-2 0.02, TLR-4 0.003, and TNF-α 0.03). Moreover, ECM regulator genes involved in tissue fibrosis and inflammation (TGF-β, α-SMA, PDGF, TIMP1, and Col1a1) were significantly overexpressed in the fibrosis group compared to those in healthy control (HC) mice (*P* < 0.0001). Principal-component analysis (PCA) was applied for the selected genes between all 25 fibrosis and 25 HC samples from seven data sets. As shown in Figure 1B, PCA indicated that the control group (blue color) was a completely separated cluster from the fibrosis group (light red color). Since these genes showed similar and significant trends in all data sets and also play a key role in liver fibrosis, this gene selection was used to study the effect of different forms of *A. muciniphila* on fibrosis-related markers. The results indicate that all eight genes were significantly differentially expressed between fibrosis groups in comparison with healthy control groups.

### Isolation and morphology of EVs.

The EVs extracted from *A. muciniphila* were evaluated by scanning electron microscopy (SEM); the results showed a spherical shape and a range in size of 40 to 150 nm (Fig. 1C). The SDS-PAGE showed protein bands covering a wide range of molecular weights, from 11 to 245 kDa, which indicates the protein content of EVs.

### Live and pasteurized *A. muciniphila* and EVs inhibited the expression of TLR-2 and TLR-4 genes in LPS-stimulated LX-2 cells.

To investigate whether *A. muciniphila* can inhibit the expression of TLR-2 and TLR-4 in LX-2 cells, we coinfected live and pasteurized *A. muciniphila* with three different multiplicities of infection (MOIs 1, 10, and 100) and EVs in various concentrations (1, 10, and 50 μg/ml) for 24 h. As shown in Figure S2, in LPS-stimulated LX-2 cells, the highest inhibitory effects of live and pasteurized *A. muciniphila* on the gene expression of TLR-2 (*P* value of <0.0001 and 0.0003, respectively) and TLR-4 (*P* value of 0.0002 and 0.001, respectively) were observed at MOI 10. In comparison with heat-killed *A. muciniphila*, which was examined in our previous study, pasteurized *A. muciniphila* showed lower mRNA level of TLR-4 at MOI 1 and 100 (Fig. S2), but the effect of heat-killed and pasteurized *A. muciniphila* was almost the same at MOI 10. EVs at a concentration of 50 μg/ml significantly (*P* < 0.0001) downregulated TLR-2 and TLR-4 expression. In the quiescent LX-2 cells, although all *A. muciniphila* treatments increased the expression of TLR-2 and TLR-4, this increase was not statistically significant (*P* value of >0.05). In quiescent cells, only EVs at a concentration of 50 μg/ml could downregulate the expression of TLR-2 gene (*P* value of 0.03). Overall, consistent with data obtained from het-killed *A. muciniphila*, these data also suggest that the effects of live and pasteurized *A. muciniphila* on quiescent and LPS-activated LX-2 cells were not dose dependent, as these two forms showed their best effect at MOI 10, while in EVs, we observed an improvement in its effect by increasing the concentration. The most effective bacterial MOIs and EVs concentration are shown in Figure 1H and I.

### EVs could efficiently induce the regression of activated HSCs.

The effect of live and pasteurized *A. muciniphila* and its EVs on gene expression of TGF-β, α-SMA, TIMP1, and Col1a1 in LX-2 cells is shown in Figure S2. In the quiescent LX-2 cells, there was no significant difference between expression of fibrosis markers in treated mice and untreated control. In LPS-stimulated LX-2 cells, the expression of fibrosis markers was dramatically increased (*P* < 0.0001). Pasteurized *A. muciniphila* could downregulate the expression of fibrosis markers at MOI 10 (*P* < 0.05), and in the case of TGF-β and α-SMA, this inhibitory effect was also seen at MOI 100 (*P* < 0.05). Almost all MOIs of live *A. muciniphila* significantly reduced the gene expression of fibrosis markers (*P* < 0.001); in the case of collagen, this reduction was observed only at MOI 10. Compared to our previous study, pasteurized *A. muciniphila* showed an mRNA level of fibrosis markers lower than that of heat-killed *A. muciniphila* (Fig. S2), but the optimum dose of heat-killed and pasteurized *A. muciniphila* was the same (MOI 10). The inhibitory effects of EVs on expression of fibrosis markers were dose dependent, and the highest antifibrosis property was observed at a concentration of 50 μg/ml (*P* < 0.0001). These observations suggest that EVs could more efficiently reverse the activation of HSCs. The most effective bacterial MOIs and EVs concentrations are shown in Fig. 1D to G.

### Oral gavage of *A. muciniphila* prevented HFD/CCL4-induced liver injury.

**FIG 2:**
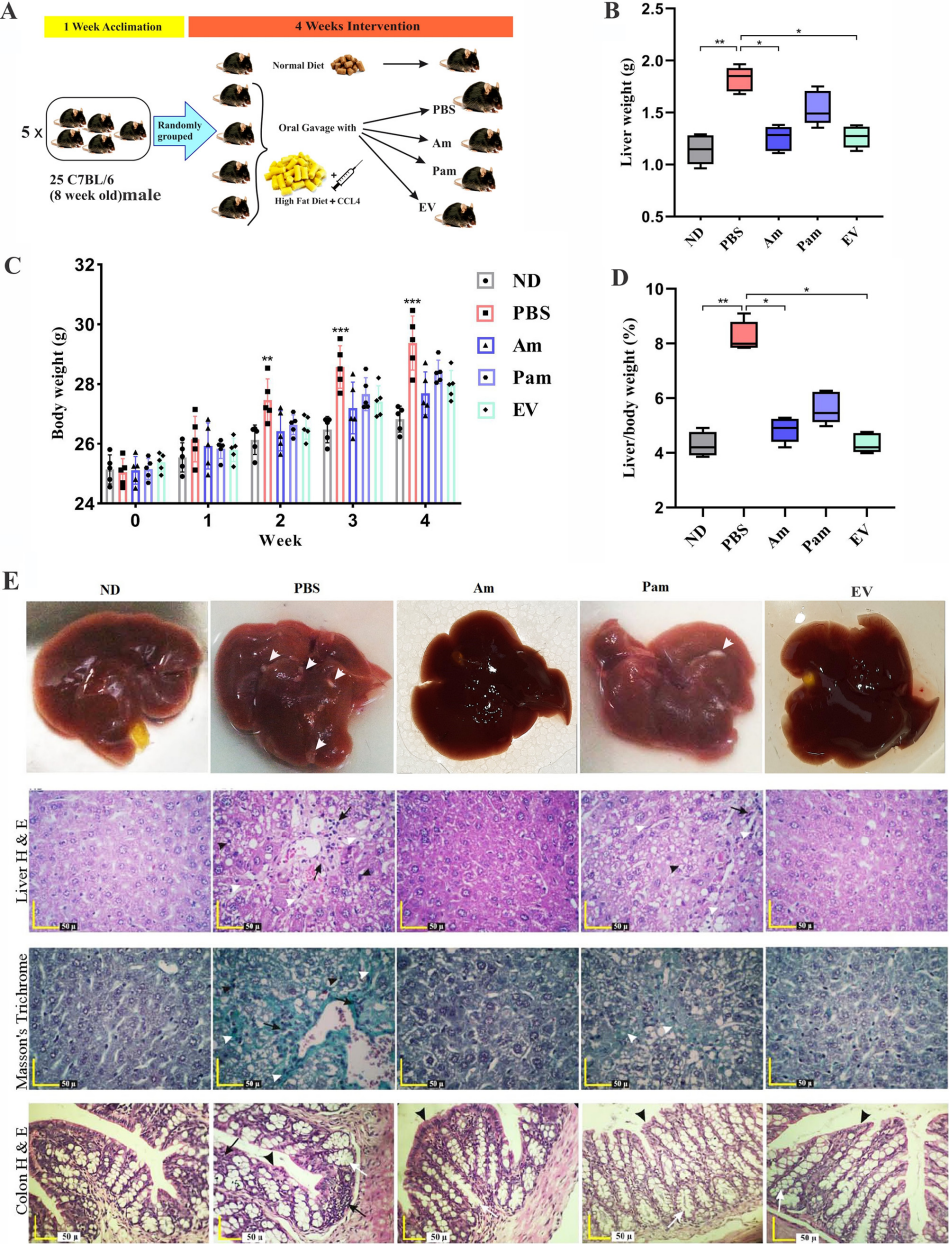
(A) Study design of the animal experiment. (B) Liver weight and (C) body weight changes were measured at the indicated time points by *post hoc* two-way ANOVA, and (D) liver/body weight ratio was measured by *post hoc* Turkey’s one-way ANOVA statistical analysis. Data are expressed as mean ± SD (*n* = 5). *, *P < *0.05 and **, *P < *0.01. (E) Representative images of gross specimens of liver, liver specimens stained with hematoxylin & eosin, liver specimens stained with Masson’s trichrome, and colon specimens stained with hematoxylin & eosin. All bacterial treatments improved liver injury and fibrosis in HFD/CCL4 mice.

In the present study, mice were treated with live *A. muciniphila* (Am), pasteurized *A. muciniphila* (Pam), and *A. muciniphila*’s extracellular vesicles (EVs) (Fig. 2A). These mice showed a mild body weight gain compared to the phosphate-buffered saline (PBS) group (*P* value of 0.025), as shown in Fig. 2B, C, and D, respectively. Although there was a trend toward improved health parameters in EV-treated mice compared to those in mice treated with live *A. muciniphila*, no significant difference was observed between the effects of these two groups on body weight (*P* value of 0.98), liver weight (*P* > 0.99), and liver/body weight ratio (*P* > 0.99). Histopathological analysis confirmed that acute liver injury was established in the PBS group compared to that in the normal diet (ND) group (Fig. 2E). Hematoxylin and eosin (H&E) and Masson’s trichrome liver staining showed that infiltration of inflammatory cells was severe in PBS and mild in pasteurized *A. muciniphila* (Pam) groups, while almost no effect of fibrosis induction was observed in live *A. muciniphila* (Am) and extracellular vesicles (EV) groups. Pericellular fibrosis occurred around damaged liver hepatocytes in the liver parenchyma, as well as around the portal portion of the liver in the PBS and pasteurized *A. muciniphila* (Pam) groups. Also, the presence of small and large droplets inside hepatocytes was observed in the PBS group compared to other groups. Moreover, we evaluated the effects of all bacterial treatments on pathology of colonic tissue. The PBS group showed the focal infiltration of inflammatory cells (including mononuclear cells, eosinophil, plasma cells, and polymorphonuclear cells) in the lamina propria and epithelium in mice colonic tissue. No inflammatory reaction was present in live *A. muciniphila* (Am) and extracellular vesicles (EV) groups. The crypt depth and thickness of the mucous layer of the colon showed a considerable decrease in the PBS group compared to that in the normal diet (ND) group. The crypt depth and thickness of the mucous layer showed an increase in pasteurized *A. muciniphila* (Pam), live *A. muciniphila* (Am), and extracellular vesicles (EV) groups compared to those in the PBS group (Fig. 2E).

### Anti-inflammatory effects of EVs.

**FIG 3:**
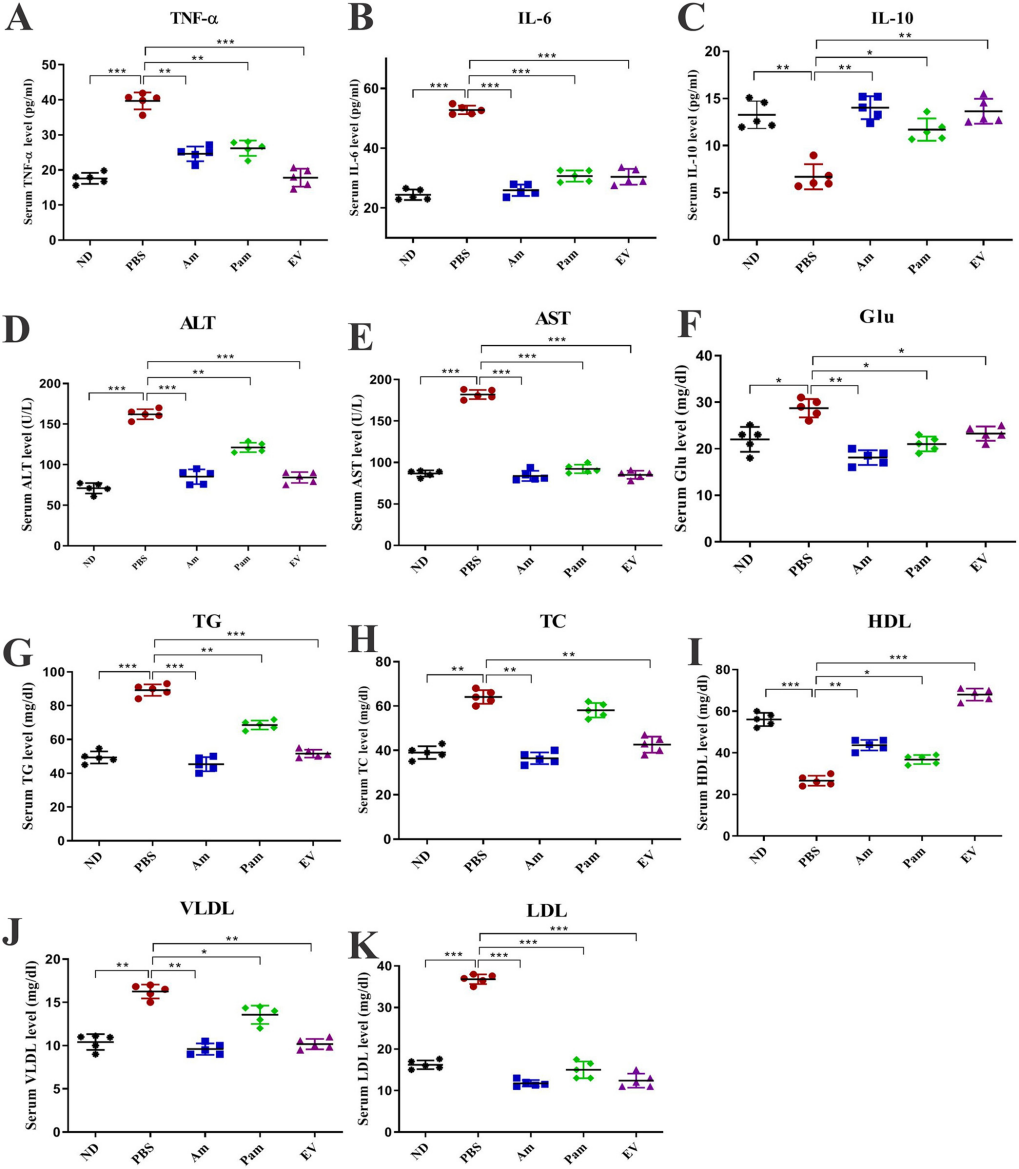
Serum biochemical and cytokines measurement. Serum levels of (A) TNF-α, (B) IL-6, and (C) IL-10; inflammatory and anti-inflammatory cytokines. Serum liver enzymes (D) ALT and (E) AST. Serum level of (F) Glu, (G) TG, (H) total cholesterol, (I) HDL, (J) VLDL, and (K) LDL. Data are expressed as mean ± SD (*n *= 5). *, *P < *0.05; **, *P < *0.01; and ***, *P < *0.001 by *post hoc* Turkey’s one-way ANOVA.

To investigate anti-inflammatory effects of live *A. muciniphila*, pasteurized *A. muciniphila*, and *A. muciniphila*’s EVs, the serum level of TNF-α, interleukin 6 (IL-6), and Il-10 was assessed. As shown in Figure 3A, B, and C, TNF-α and IL-6 significantly increased (*P* < 0.0001) and IL-10 significantly decreased (*P* value of 0.003) in PBS compared to those in the ND group. Interestingly, all treatments could significantly decrease serum level of TNF-α (*P* value of <0.0001 for EVs, 0.001 for Am and Pam) and IL-6 (*P* < 0.0001) while increasing IL-10 (*P* value of 0.001 for EVs, 0.002 for Am, and 0.01 for Pam). Interestingly, these anti-inflammatory effects were more pronounced in EV than in other groups, suggesting that *A. muciniphila* may inhibit liver fibrosis through modulating of the serum cytokines, which is more evident in EVs.

### *A. muciniphila* improved serum biochemical and liver enzymes in HFD/CCL4-induced liver injury mice.

Evaluation of serum levels of liver enzymes showed a significant increase in alanine aminotransferase (ALT) and aspartate aminotransferase (AST) levels of the PBS group compared to those of the ND group (*P* < 0.0001). Daily oral gavage with all bacterial treatment was able to decrease serum level of ALT and AST, while in Am and EV groups, this improvement was much more pronounced (*P* < 0.0001) than that in the Pam group. As shown in Figure 3D to K, HFD/CCL4 caused a significant increase in the serum level of Glu (*P* value of 0.01), total cholesterol (TC; *P* value of 0.001), total triglyceride (TG; *P* < 0.0001), low-density lipoprotein (LDL; *P* < 0.0001), and very-low-density lipoprotein (VLDL; *P* value of 0.002), while in the serum level of high-density lipoprotein (HDL; *P* < 0.0001), it had the opposite effect in the PBS group compared to that in the ND group. Administration of two forms of whole cell bacteria and EVs reduced blood glucose, especially in the Am group (live bacteria) compared to that in the PBS group (*P* value of 0.002). Also, a significant decrease in the serum level of TG and TC was observed in Am and EV in comparison with that in the PBS groups (*P* value of <0.001 and 0.001, respectively), whereas TC level did not show a significant difference in the Pam group compared to that in the PBS group (*P* value 0.07), and the effect of *A. muciniphila* on TG was also less significant than that of the other forms used in this study (*P* value of 0.003). In the case of LDL, all bacterial forms and EVs significantly (*P* < 0.0001) decreased the LDL compared to that of the PBS group. Although VLDL was significantly reduced in all groups, Am and EV showed the highest decrease compared to PBS (*P* value of 0.001 and 0.003, respectively). Surprisingly, the highest incremental effect on HDL was related to EV between the groups (*P* < 0.0001). Overall, these results suggest that *A. muciniphila* and its EVs could prevent liver fibrosis by normalizing serum glucose, lipid profiles, and liver enzymes.

### Treatment with EVs and *A. muciniphila* decreased fibrosis markers in mice liver tissue.

**FIG 4:**
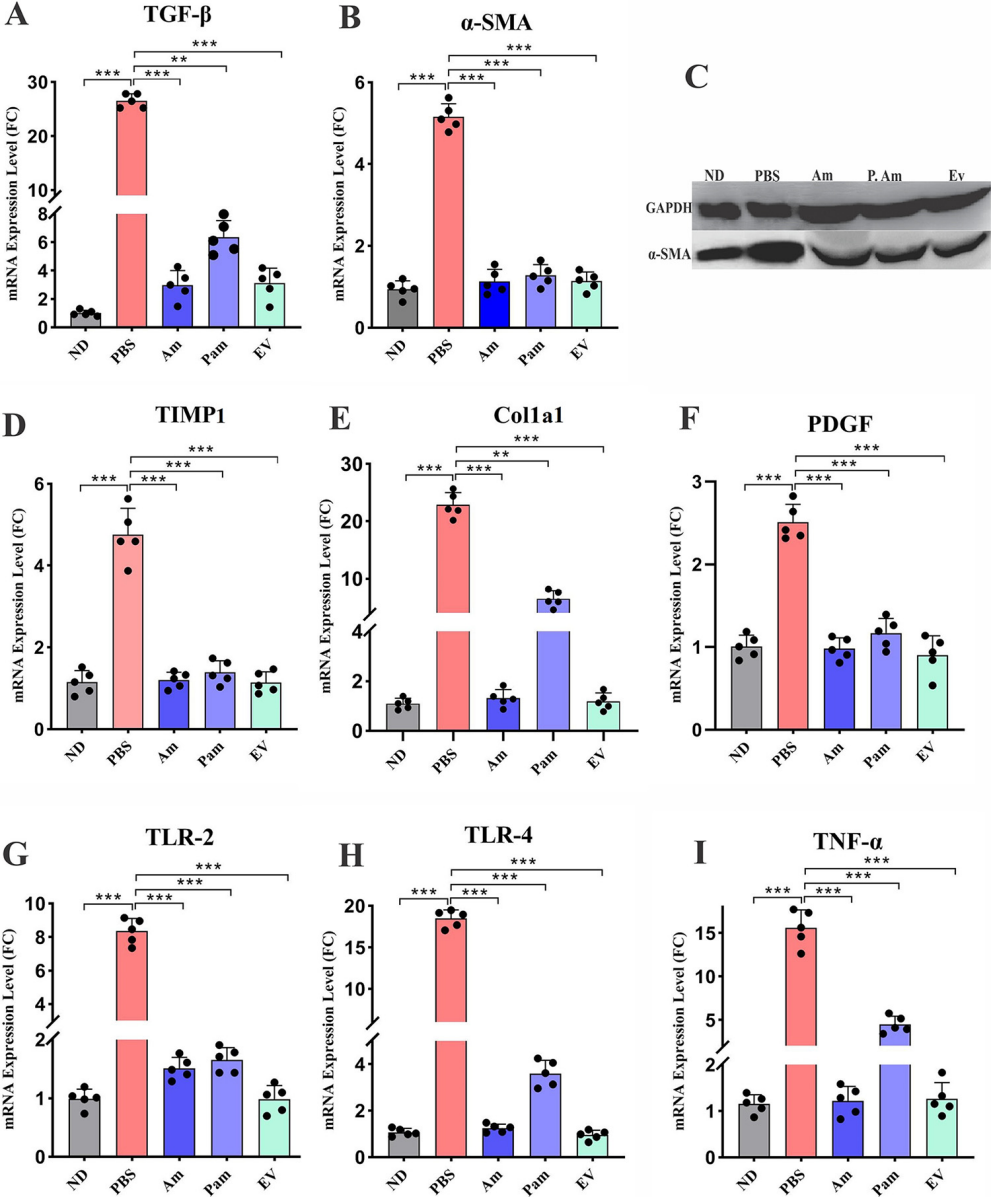
Hepatic mRNA expression of liver fibrosis-related genes (A) TGF-β, and (B) α-SMA. (C) Western blotting of α-SMA. Hepatic mRNA expression of liver fibrosis-related genes (D) TIMP1, (E) Col1a1, and (F) PDGF. Hepatic mRNA expression of inflammatory-related genes in mice liver tissue, (G) TLR-2, (H) TLR-4, and (I) TNF-α. Data are expressed as mean ± SD (*n *= 5). *, *P < *0.05; **, *P < *0.01; and ***, *P < *0.001 by *post hoc* Turkey’s one-way ANOVA.

To explore the protective effects of *A. muciniphila* and its EV on liver fibrosis, we assessed gene expression of fibrosis markers on liver tissue of mice. As shown in Figure 4, HFD/CCL4 significantly upregulated the gene expression of fibrosis markers in the PBS group compared to that in the ND group (*P* < 0.0001). The gene expression analysis showed that live *A. muciniphila* and its EVs modulated the expression of α-SMA, PDGF, TIMP1, TGF-β, and Col1a1 genes (*P* < 0.0001). As demonstrated in Figure 4, the inhibitory effect of Pam on Col1 and TGF-β was less significant than that on two other groups (*P* value of 0.001). These results suggest that administration of live and pasteurized *A. muciniphila* and its EVs could reduce hepatic damage induced by HFD/CCl4 through inhibiting the expression of α-SMA, PDGF, TIMP, and Col1a1 genes.

### *A. muciniphila* and its EVs prevented liver fibrosis through reducing inflammation.

To investigate the influence of *A. muciniphila* on the inflammatory status of liver tissue, we assessed the expression of TLR-2, TLR-4, and TNF-α genes in HFD/CCl4 mice. A significant increase was observed in gene expression of these inflammatory markers in the PBS group compared to that in the ND group (*P* < 0.0001). As shown in Figure 4, the mRNA expression level of these genes was significantly reduced in all bacterial forms compared to that in the PBS group (*P* < 0.0001). This inhibitory effect was similar in all study groups, indicating that various forms of *A. muciniphila* could downregulate the expression of inflammatory genes in liver tissue and consequently inhibit hepatic injury induced by HFD/CCl4.

### Administration of *A. muciniphila* and its EVs improved intestinal barrier integrity in HFD/CCL4-treated mice.

**FIG 5:**
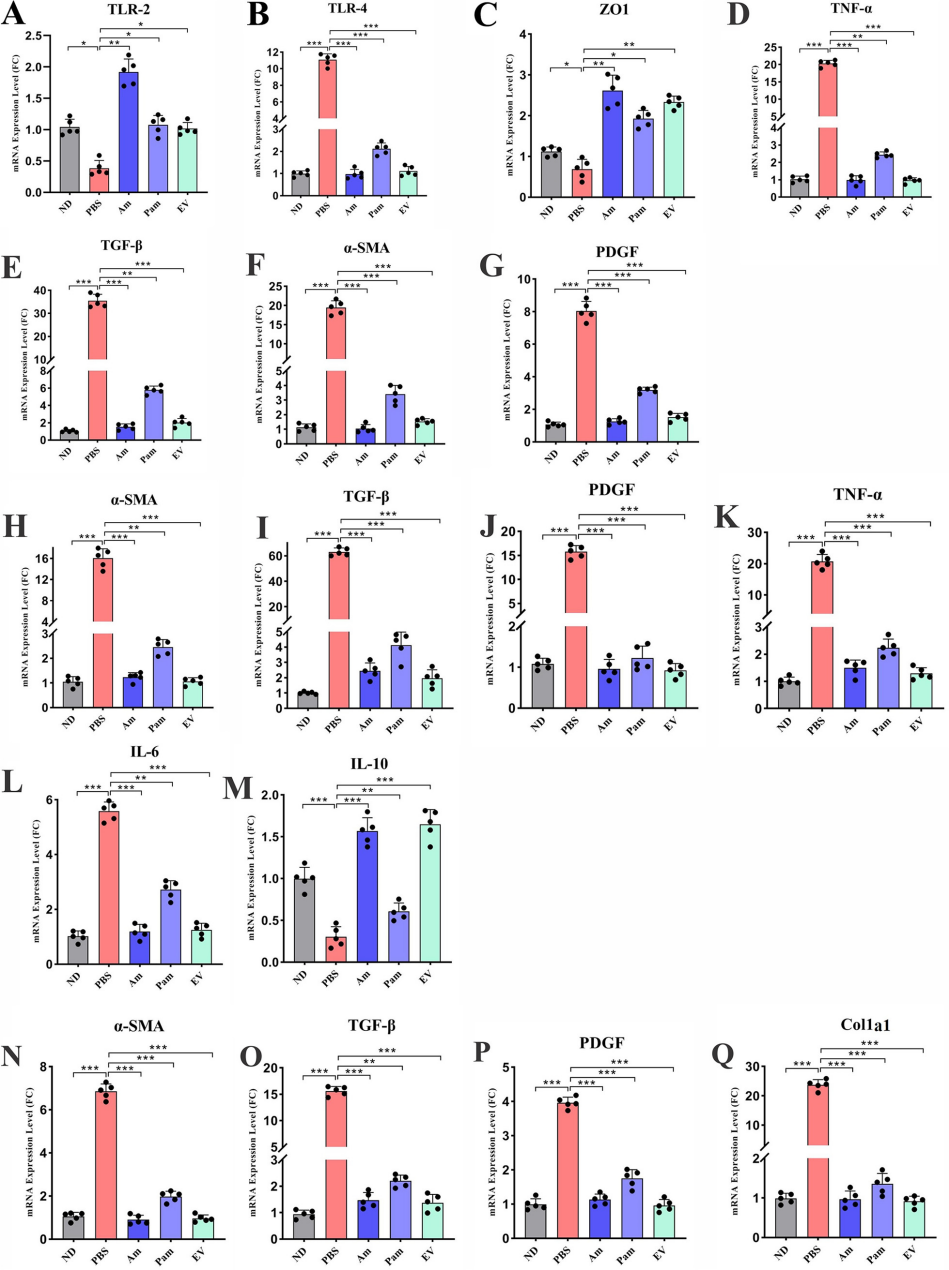
All *A. muciniphila* supplementation reduced inflammatory- and fibrosis-related mRNA expression levels in colon, adipose, and kidney tissue. Relative mRNA levels of different inflammatory and fibrosis markers in colon tissue, (A) TLR-2, (B) TLR-4, (C) ZO1, (D) TNF-α, (E) TGF-β, (F) α-SMA, and (G) PDGF. Relative mRNA levels of inflammatory and fibrosis markers in adipose tissue, (H) α-SMA, (I) TGF-β, (J) PDGF, (K) TNF-α, (L) IL-6 and (M) IL-10. Relative mRNA levels of fibrosis markers in kidney tissue, (N) α-SMA, (O) TGF-β, (P) PDGF, and (Q) Col1a1. Data are expressed as mean ± SD (*n *= 5). *, *P < *0.05; **, *P < *0.01; and ***, *P < *0.001 by *post hoc* Turkey’s one-way ANOVA.

The colon is the first place to be affected by HFD, dysbiosis, inflammation, and impaired intestinal permeability, which lead to systemic inflammation and eventually liver involvement. Since an increase in the intestinal mucosal permeability and inflammation directly affects liver dysfunction, ZO-1, TLR-2, TLR-4, and TNF-α expression was assessed in the colon tissue of HFD/CCL4-treated mice. As shown in Figure 5B and D, administration of various forms of *A. muciniphila* and its EVs could enhance the expression of ZO-1 mRNA in the colon tissue (*P* value of 0.001 for Am, 0.003 for EV, and 0.043 for Pam), while in the PBS group it was significantly downregulated (*P* value of 0.01). The TLR-2 was another factor that was upregulated in all treatment groups compared to that in PBS; however, the highest increase was observed in the Am group (*P* value of 0.002 in Am and 0.03 for Pam and EV) (Fig. 5A). The expression of TLR-4 and TNF-α was significantly downregulated in all treatment groups in comparison with that in the PBS group (*P* < 0.0001) (Fig. 5C). It can be implied that oral gavage with live and pasteurized *A. muciniphila* and its EVs is able to improve the function of intestinal barrier and ameliorate the inflammatory response in the colon and possibly induce intestinal immune hemostasis.

### Antifibrosis effects of *A. muciniphila* in the colonic tissues of HFD/CCL4-treated mice.

In this study, the gene expression data showed that HFD along with CCL4 injection resulted in overexpression of α-SMA, PDGF, and TGF-β genes in the PBS group (*P* < 0.0001). Interestingly, we found that the all forms of *A. muciniphila* and its EVs had an inhibitory effect on these fibrosis gene markers in the colon. However, live *A. muciniphila* and its EVs downregulated the expression of α-SMA and TGF-β (*P* < 0.0001) more significantly than did the pasteurized form (*P* value of 0.001). In the case of PDGF, all forms of *A. muciniphila* and its EVs demonstrated significant inhibitory effects (*P* < 0.0001). Overall, the results suggested that *A. muciniphila* and EVs could downregulate the fibrosis markers not only in the liver but also in the colon tissue (Fig. 5E to G).

### *A. muciniphila* and its EVs ameliorated inflammation and fibrosis in epididymal white adipose tissue.

In chronic liver injury, adipose tissue is inflamed and displays the features of dysfunction; thus, in order to investigate this issue, we examined the expression of inflammatory and fibrosis genes in the mice epididymal white adipose tissue (eWAT). In the PBS group, fibrosis genes, including α-SMA, PDGF, and TGF-β, and proinflammatory genes, such as TNF-α and IL-6, were overexpressed (*P* < 0.0001), while the anti-inflammatory IL-10 was decreased (*P* < 0.0001). Our results demonstrated that live *A. muciniphila* and its EVs could downregulate the gene expression of α-SMA and IL-6 and upregulate the IL-10 (*P* < 0.0001) more significantly than could pasteurized *A. muciniphila* (*P* value of 0.001, 0.004, and 0.002, respectively) (Fig. 5H to M). These results showed that the use of *A. muciniphila* not only improved the liver and colon functions but also showed promising antifibrosis and anti-inflammatory effects in eWAT.

### Antifibrotic effect of *A. muciniphila* and its EVs on kidney.

In order to investigate whether the EVs or various forms of *A. muciniphila* could have antifibrosis activities on the kidney tissue, we evaluated α-SMA, PDGF, Col1a1, and TGF-β mRNA expression levels between study groups. As demonstrated in Fig. 5N to Q, CCl4 administration markedly increased the expression of these genes in the PBS group (*P* < 0.0001). However, although all bacterial treatments significantly reduced the gene expression of these markers (*P* < 0.0001), the impact of pasteurized *A. muciniphila* on TGF-β downregulation was less significant than that of other treatment groups (*P* value of 0.001). Overall, the results showed that all three bacterial forms used had a significant preventive effect on the HFD/CCL4-induced injury in kidney tissue.

### Reduction of pathobionts after using live *A. muciniphila*.

**FIG 6:**
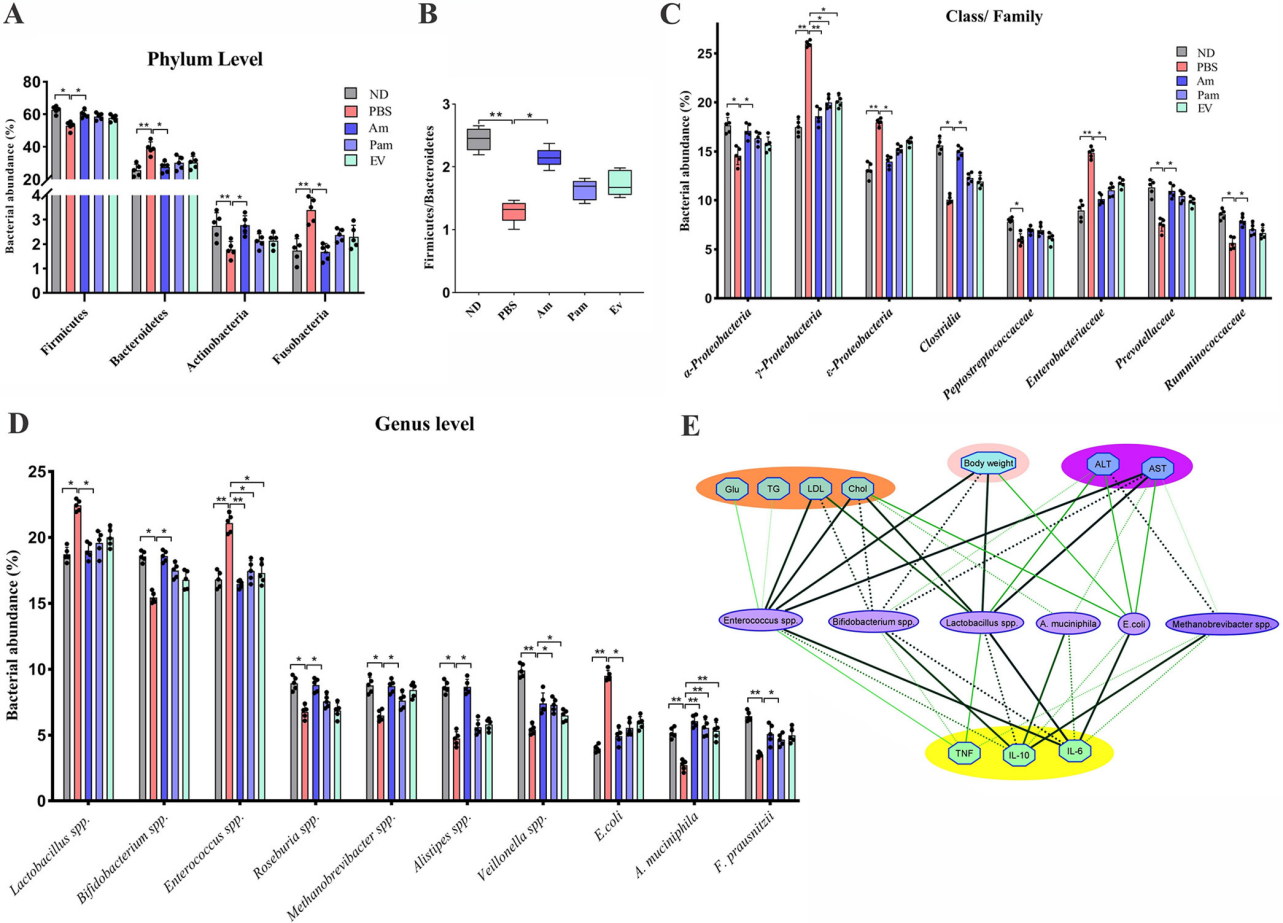
The relative percentage of targeted gut microbiome. Data are expressed as mean ± SD (*n* = 5). (A) Gut microbiome composition at the phylum level. (B) Ratio of *Firmicutes* to *Bacteroidetes*. (C) Gut microbiome composition at the class/family level. (D) Gut microbiome composition at the genus level. *, *P < *0.05; **, *P < *0.01 by nonparametric Kruskal–Wallis test. (E) Correlation analysis between targeted gut microbiota and biological indices. Spearman’s rho nonparametric correlation was used, and significant relationships with *P *value of <0.05 and *r-*rank of >0.5 are shown. Blue nodes: biological indices. Purple nodes: differentially distributed genera between groups; the thickness of the connection represents the correlation.

Since an HFD affects gut bacterial composition and induces intestinal dysbiosis, the effect of live, pasteurized *A. muciniphila* and its EVs on the intestinal microbial community was investigated (Fig. 6A). HFD/CCL4 administration significantly decreased *Firmicutes* (*P* value of 0.05) and increased *Bacteroidetes* (*P* value of 0.01) relative abundance in the PBS group compared to that in the ND group (55.54% versus 37.88% in PBS group, 65.13% versus 29.7% in ND group), while administration of live *A. muciniphila* could considerably restore this imbalance (61.67% for *Firmicutes* versus 31% for *Bacteroidetes*). The effect of pasteurized *A. muciniphila* and its EVs on *Firmicutes* and *Bacteroidetes* abundance was not statistically significant (59.1% versus 31.74% for Pam group and 0.14%; 59.04% versus 35.34% for EV group). The *Firmicutes* to *Bacteroidetes* (F/B) ratio was significantly (*P* value of 0.01) lower in the PBS group than in the ND group. In addition, the F/B ratio in the Am group was significantly (*P* value of 0.05) higher than that in the PBS group. As shown in Figure 6B, the F/B ratio in pasteurized *A. muciniphila* and EV groups was higher than that in the PBS group, but this difference was not statically significant. Moreover, HFD/CCl4 significantly increased the relative abundance of *Fusobacteria* and decreased *Actinobacteria* in the PBS group compared to those in the ND group (*P* value of 0.01 and 0.02, respectively), while only live *A. muciniphila* could significantly balance these two phyla (*P* value 0.04 and 0.03, respectively). As shown in Figure 6C, a significant increase was observed in the relative abundance of *Gammaproteobacteria* (*P* value of 0.01), *Epsilonproteobacteria* (*P* value of 0.01), and *Enterobacteriaceae* (*P* value of 0.025) at the class/family level in the PBS group, while live *A. muciniphila* was able to significantly increase *Clostridia* (*P* value of 0.02), *Ruminococcaceae* (*P* value of 0.02), and *Prevotellaceae* (*P* value of 0.03). At the genus/species level, *Lactobacillus* spp. and Escherichia coli were enriched in PBS compared to those in the ND (*P* value of 0.03 and 0.01, respectively), while *Bifidobacterium* spp., *Roseburia*, *Methanobrevibacter*, *Alistipes*, *Veillonella*, and Faecalibacterium prausnitzii were more abundant in the ND group (*P* value of 0.03, 0.02, 0.03, 0.04, 0.01, and 0.01, respectively) and the Am group (*P* value of 0.05, 0.02, 0.03, 0.02, 0.03, and 0.04, respectively) than in the PBS group (Fig. 6D). As expected, the relative percentage of *A. muciniphila* in all treated groups was higher than that in the PBS group (*P* value of 0.01), which indicated that live bacteria colonization occurred successfully after 4 weeks of oral gavage. The pasteurized *A. muciniphila* and its EVs also possibly induced the *A. muciniphila* growth after oral gavage. The pasteurized *A. muciniphila* could also modify the intestinal targets microbiota of mice in the case of *Gammaproteobacteria*, *Veillonella*, and *Enterococcus* (*P* value of 0.04, 0.03, and 0.03, respectively). Moreover, the EVs positively altered the mice intestinal targets’ microbiota compared to that of the PBS group in the case of *Gammaproteobacteria* and *Enterococcus* (*P* value of 0.04 and 0.04, respectively); however, other targets’ microbiota changes were not statistically significant. These data demonstrate the ability of live *A. muciniphila* to reduce the relative abundance of pathobionts and increase symbionts, therefore shifting the targets’ microbiome structures to be similar to those of the ND group.

### *A. muciniphila* modulated host responses to HFD/CCl4-induced liver injury by normalizing mice targeted gut microbiome.

To further explain the impact of gut microbiome structure, we adopted correlation analysis between the relative abundance of bacteria at the genus/species level assessed in this study and blood biomarkers. As shown in Figure 6E, *Enterococcus* spp., which were more abundant in the PBS group, positively correlated with serum level of TG (*r*-rank of 0.88, *P* value of 0.04), TC, LDL, and Glu (*r*-rank of 0.98, 0.97, and 0.88, *P* value of 0.003, 0.005, and 0.04, respectively). The serum level of TC and LDL was also positively correlated with *Lactobacillus* spp. (*r*-rank of 0.97 and 0.94, *P* value of 0.006 and 0.01, respectively). Moreover, Escherichia coli showed positive association with TC (*r*-rank of 0.9, *P* value of 0.03). Conversely, TC was negatively associated with beneficial bacteria such as *A. muciniphila* and *Bifidobacterium* spp. (*r*-rank of −0.90 and −0.97, *P* value of 0.03 and 0.004, respectively), which were more abundant in the ND and Am groups. *Bifidobacterium* spp. also showed negative association with LDL (*r*-rank of −0.96, *P* value of 0.007). *Enterococcus* spp. (*r*-rank of 0.97 for IL-6 and 0.88 for TNF-α, *P* value of 0.04 and 0.004, respectively), *Lactobacillus* spp. (*r*-rank of 0.99 for IL-6, *P* value of 0.001), and E. coli (r-rank 0.97 for IL-6, *P* value 0.005) also demonstrated positive correlation with serum proinflammatory cytokines, while they inversely associated with anti-inflammatory cytokine IL-10 (*r*-rank of −0.93, −0.96, and −0.90, *P* value of 0.02, 0.008, and 0.03, respectively). ALT, as one of the most important blood biomarkers in liver injury, was negatively related to *Methanobrevibacter* and *Bifidobacterium* spp. (*r*-rank of −0.89 and −0.90, *P* value of 0.003 and 0.04, respectively) and was positively associated with E. coli and *Lactobacillus* spp. (*r*-rank of 0.90 and 0.91, *P* value of 0.03 and 0.03, respectively). Another liver enzyme, AST, was inversely related to *Methanobrevibacter* and *Bifidobacterium* spp. and *A. muciniphila* (*r*-rank of −0.87, −0.99, and −0.89, *P* value of 0.04, 0.001, and 0.03, respectively), while it was positively related to *Enterococcus* spp., *Lactobacillus* spp., and E. coli (*r*-rank of 0.99 and 0.90, *P* value of 0.001 and 0.03, respectively). Likewise, the body weight also demonstrated positive correlation with *Enterococcus* spp., *Lactobacillus* spp., and E. coli (*r*-rank of 0.98, 0.97, and 0.90, *P* value of 0.003, 0.005, and 0.03, respectively), while it demonstrated negative correlation with *Bifidobacterium* spp. (*r*-rank of −0.97, *P* value of 0.004). These data proposed the beneficial impact of gut microbiota on modulating the host response against the HFD/CCl4 challenge.

## DISCUSSION

As a fundamental pathological indicator of chronic liver injury, fibrosis has recently received more attention due to its reversible characteristics (31). Given the absence of successful therapies for liver fibrosis and blind treatment of most patients, new preventive strategies are urgently needed for the management of liver fibrosis (32). *A. muciniphila*, as a next-generation probiotic with beneficial effects on the host metabolic system and immune responses, has been introduced as a promising therapeutic option for microbiota-related diseases (33). So far, the positive effects of *A. muciniphila* and its products have been explored in a number of studies, and various disorders seem to be associated with the disturbance of its abundance in the gut (20, 34, 35). Nevertheless, due to the possible risks of live bacterial cells, more attention has been paid to the use of nonviable bacteria with bioactivity, known as “paraprobiotics” and “postbiotics” (36).

In our previous work, we observed that administration of *A. muciniphila* and its EVs was associated with weight loss and lower fat mass in the treatment of HFD-induced obese mice by affecting satiety hormones and controlling appetite (37). Therefore, the primary goal of the present study was to determine whether live and pasteurized forms of *A. muciniphila* and its EVs could inhibit liver fibrosis in an HFD/CCl4-induced mouse model. We analyzed the antifibrotic and anti-inflammatory effects of *A. muciniphila* in the liver, colon, eWAT, and kidney tissues of HFD/CCl4 mice. Our results demonstrated the deterrent effects of live and pasteurized *A. muciniphila* and its EVs on liver damage, in addition to the improvement of liver and colon histopathology in HFD/CCl4-fed mice. According to previous studies, liver damage is closely related to sex, and an increase in male liver sensitivity to injury may be related to the gonad endocrine (38, 39). In addition, the benefits of estrogen in liver injury repair have been well established in animal models (38). Therefore, the use of male mice model to induce liver fibrosis in the present study can be justified. We also observed that oral gavage with *A. muciniphila* and its EVs could reduce weight gain compared with that of the PBS group, while the pasteurized form had less of an effect on body weight compared to that of two other treatment groups. Moreover, this lower increase in body and liver weight in Am and EV groups was associated with a decrease in liver damage in histopathological examinations of liver tissues. Another study found that gavage with a similar amount of *A. muciniphila* for 5 weeks reduced weight gain and visceral fat size and inhibited insulin resistance in HFD mice (40). Considering the effects of *A. muciniphila* on reducing insulin resistance, as well as the effect of its EVs on satiety hormones and appetite control, it can be said that one of the possible mechanisms by which this bacterium and its derivatives could prevent the development of liver fibrosis in the HFD/CCl4 mouse model is through controlling weight gain in the study groups.

Serum levels of transaminases (ALT and AST) are associated with the severity of liver histopathology. Evidence shows that an elevated ALT/AST level can result in a higher grade of liver injury (41). Systemic inflammation is another key indicator of the pathogenesis of liver fibrosis, and IL-6, TNF-α, and TGF-β are the main cytokines in this pathological condition (42, 43). Our results also showed that the serum levels of ALT and AST dramatically increased in HFD/CCl4-fed mice, while all forms of *A. muciniphila* could adjust the serum levels of transaminases, as well as those of Glu and lipid profiles (TG, TC, LDL, VLDL, and HDL). This serum biochemical adjustment was also associated with the decreased level of proinflammatory cytokines (IL-6 and TNF-α) and increased production of anti-inflammatory cytokines (IL-10).

Recently, Kim et al. reported that oral administration of *A. muciniphila* for 10 weeks could reduce the serum TG and ALT levels in HFD-fed obese mice (44). Also, Grander et al. observed that three doses of 1.5 × 10^9^ CFU *A. muciniphila* could decrease hepatic injury, restore the intestinal barrier integrity, alleviate the serum ALT level, and diminish the expression of TNF-α in a mouse model of alcohol-induced liver injury (45). Another study also showed that daily administration of live *A. muciniphila* for 5 weeks could modulate TG, Glu, insulin, LPS-binding protein (LBP), and leptin and reduce the fat mass in HFD-fed mice (40). Moreover, Wu et al. proposed that the *A. muciniphila* MucT strain had beneficial effects on acute immune-mediated liver injury in a mouse model of concanavalin A-induced liver injury by attenuating the serum levels of various proinflammatory cytokines, ALT, and AST over 14 days (46). Also, Yang et al. demonstrated the protective activity of pasteurized *A. muciniphila* against HFD-induced inflammation in mice, which was characterized by the reduced production of colonic TNF-α and IL-6 and increased secretion of IL-10 (47). Therefore, our findings not only confirmed the results of previous studies but also showed that nonviable pasteurized *A. muciniphila* and its EVs could prevent HFD/CCL4-induced liver injury through regulation of various serum markers.

HSCs play a crucial role in the progression and reversion of liver fibrosis (48). Activated HSCs are characterized by the expression of α-SMA and excessive accumulation of ECM proteins through inflammatory cytokines, which promote the progression of fibrosis (49). Moreover, it has been documented that inactivation of HSCs significantly affects the convergence of immune responses and inhibits fibrogenesis (50). In the current study, we demonstrated that live and pasteurized *A. muciniphila* and its EVs had inhibitory effects on the HSC activation; the EVs showed the highest HSC restoring effect in the LX-2 cells.

In the present study, we also confirmed our previous findings, as heat-inactivated *A. muciniphila* attenuated HSC activation in LPS-stimulated LX-2 cells (15). Our results showed that EVs could significantly reduce the relative expression of fibrosis and inflammatory markers in the liver, eWAT, and kidney tissues of mice. Since *A. muciniphila* EVs can transmit through epithelial and endothelial barriers, they may indirectly interfere with HSC activation and subsequently ameliorate the hepatic injury. In agreement with our findings, previous studies have also shown that Lactobacillus rhamnosus GG and selenium-glutathione-enriched probiotics reduced the liver fibrosis markers and prevented liver injury (51, 52).

The bidirectional interaction of the gut and liver, known as the gut-liver axis, is established through the portal vein, which directly transports gut-derived products to the liver; the liver feedback is the release of bile and antibodies in the intestine (53). Over the past few years, the gut dysbiosis has been argued to be an important phenomenon in the progression of liver diseases (54). On the other hand, recent studies have suggested that modulation of the intestinal microbiota by probiotic supplementation could be used as a promising therapeutic option for liver diseases (51, 52, 55).

Extensive research has confirmed that oral administration of live *A. muciniphila* promotes microbiota diversity, induces anti-inflammatory responses, modulates metabolic signaling, and leads to immune tolerance in the gut and other organs (45, 46, 56). In this regard, a randomized double-blind trial showed that a 6-week intervention with unpasteurized and pasteurized lacto-fermented bacteria altered the gut microbiota and reduced the severity of irritable bowel syndrome (IBS) (57). Conversely, another study showed that heat-inactivated Lactobacillus fermentum and Lactobacillus delbrueckii could weakly alter the gut microbial community at the genus level in healthy mice (58). Our results also showed that live *A. muciniphila* could successfully alter the intestinal microbiota in favor of a beneficial bacterial population and reduce pathobionts. This finding indicates that *A. muciniphila* may also have indirect inhibitory effects on liver fibrosis by balancing the structure of intestinal microbiota and maintaining its homeostasis.

On the other hand, we observed that pasteurized *A. muciniphila* and its EVs could not entirely normalize the intestinal microbiota composition in the studied mice. This finding supports the beneficial effects of live bacterial cells of *A. muciniphila* in regulation of the gut microbiome, mainly through the colonization resistance phenomenon and production of various metabolites, such as short-chain fatty acids (SCFAs) in the intestinal environment (59). In addition, *A. muciniphila* is known as one of the few bacterial species capable of using mucin as a sole energy source and releases products that are used by butyrate-producing bacteria. Because of this mucin-degrading ability, it is believed that this bacterium is a keystone species in the healthy gut (60). Furthermore, a recent study shows that live *A. muciniphila* had major effects on metabolism in the gut and the liver, while the pasteurized *A. muciniphila* was more effective in elevating the beneficial metabolites, especially in the gut of antibiotic treated mice (61). However, the exact mechanism underlying the effectiveness of *A. muciniphila* and its derivatives in a healthy gut microbiota population remains to be elucidated, particularly by using different animal models such as germ-free or *A. muciniphila*-decontaminated mice.

The main pathological mechanism underlying bacterial translocation is the impaired intestinal epithelial integrity due to the reduction of tight-junction proteins, which results in the induction of endotoxemia, as seen in the pathophysiology of ALD and NASH (62). Furthermore, the liver is the first organ that responds to bacterial flagellin and LPS by activating the TLRs signaling pathways (63). Likewise, the gut dysbiosis promotes local chronic inflammation and dysfunction of the intestinal permeability (64). Intestinal fibrosis is also another consequence of local chronic inflammation, characterized by the excessive deposition of ECM proteins in the colonic tissue (65). The results of this study indicated that administration of live *A. muciniphila* exerted protective effects on the intestinal epithelial integrity by upregulation of ZO-1, in addition to downregulation of intestinal fibrosis markers and inflammatory markers. Although pasteurized *A. muciniphila* and its EVs had beneficial effects on the intestinal integrity and fibrosis prevention, their positive effects were less significant than those of live bacteria. Similarly, several studies showed that oral administration of *A. muciniphila* and/or its EVs could ameliorate intestinal inflammation, regulate tight-junction proteins, and consequently improve the intestinal permeability (20, 37, 66). In this regard, Imai et al. observed a relationship between the gut dysbiosis and the risk of intestinal fibrosis, suggesting that treatment of the gut dysbiosis with probiotics could be a novel therapeutic approach to prevent intestinal fibrosis (67). Moreover, according to a comparative study of two *A. muciniphila* strains (ATCC BAA-835 and strain 139), the ATCC strain (similar to the strain of the present study) was associated with an increase in the production of SCFAs in a dextran sulfate sodium (DSS)-induced chronic murine colitis model, although both strains showed anti-inflammatory effects and normalized the gut microbiota (66).

Dysfunction of gastrointestinal barriers can also be a potential factor in adipose tissue inflammation due to macrophage activation and production of proinflammatory markers, such as IL-6 and TNF-α (68). In addition, the adipose-liver axis represents another interorgan relationship, which is mediated by signaling molecules, such as adipokines, cytokines, and damage-associated molecular patterns (DAMPs), released by the adipose tissue into the portal vein (69). An improper cross talk between the adipose and liver tissues promotes insulin resistance, liver inflammation, and development of steatohepatitis (70). Moreover, adipose tissue fibrosis, which is characterized by collagen accumulation, contributes to the transition of free fatty acids to the liver and leads to the development of hepatic steatosis and NAFLD (71).

Our results showed that EVs had stronger anti-inflammatory and antifibrotic effects on the eWAT of HFD/CCl4-treated mice than did live and pasteurized forms of *A. muciniphila*. The enhanced effect of EVs is probably due to their ability to diffuse into the eWAT and exert direct regulatory effects, while bacterial cells seem unable to affect the adipose tissue directly. We also confirmed our previous finding, which showed that *A. muciniphila* exerted anti-inflammatory effects in the eWAT of HFD-induced obese mice by increasing the expression of peroxisome proliferator-activated receptor alpha/gamma (PPAR-α/γ) and downregulating TGF-β, while the effect of EVs was more noticeable (16).

Furthermore, Schneeberger et al. reported that the decreased abundance of *A. muciniphila* was strongly associated with the expression of lipid metabolism and inflammatory markers in the adipose tissues of mice (72). Recently, Depommier et al. showed that pasteurized *A. muciniphila* significantly increased the energy expenditure and decreased the total adipose depot weight in HFD mice compared to those in ND mice (73). However, it is worth mentioning that in the study by Deng et al., only a specific strain of *A. muciniphila* (Amuc-GP01) improved glucose tolerance, hyperlipidemia, and liver steatosis in HFD-fed mice (74). Overall, accumulating evidence suggests that *A. muciniphila* not only improves the liver and colon functions but also induces potent antifibrosis and anti-inflammatory effects in the adipose tissue.

Liver dysfunction is also associated with an increased risk of incident chronic kidney disease (CKD) (75). Dysbiosis and impaired integrity of the intestinal barrier can lead to an altered microbial metabolism, insulin resistance, systemic inflammation, and activation of profibrogenic factors (76). These biological events contribute to the progression of liver dysfunction and trigger pathways that influence the development of kidney injury (77). On the other hand, kidney damage can elevate the level of uremic toxins in the systemic circulation, which in turn induces gut dysbiosis and its consequences that trigger liver dysfunction (78). In the present study, we showed that live and pasteurized *A. muciniphila* and its EVs could decrease the expression of α-SMA, Col1a1, TGF-β, and TIMP1 genes in the kidney tissues of mice. This beneficial effect of *A. muciniphila* on the kidney may be a result of induced hemostasis and interorgan cross talk in the HFD/CCl4-fed mice.

Despite the beneficial effects of *A. muciniphila* reported in this study, further in-depth investigations are required to identify the mechanisms through which *A. muciniphila* may promote the kidney function and health. Consistent with our results, Yang et al. (79) demonstrated that administration of a mixture of *Lactobacillus* strains alleviated CKD and leaky gut and decreased apoptosis; consequently, it suppressed systemic inflammation and kidney fibrosis in mice undergoing nephrectomy. Moreover, a recent systematic review and meta-analysis of randomized controlled trials revealed that probiotic, prebiotic, and synbiotic supplements ameliorated inflammatory biomarkers, oxidative stress, and lipid profiles among patients with CKD (80). However, based on the results of another systematic review and meta-analysis by McFarlane et al. (81), the supplementary effects of prebiotics, probiotics, and synbiotics on the clinical outcomes of CKD were uncertain, and there was limited information to validate these beneficial effects.

**FIG 7:**
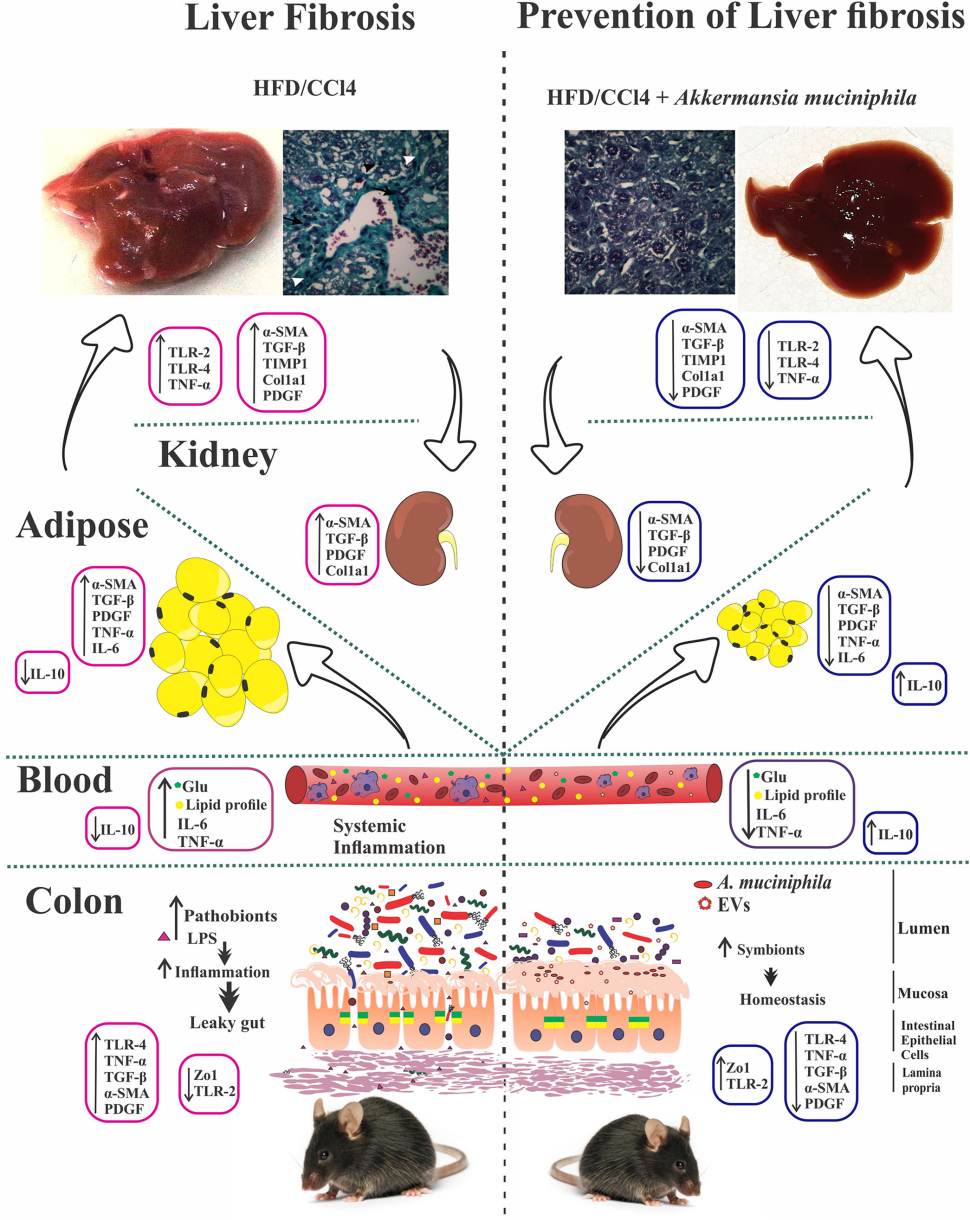
Oral administration of different forms of *A. muciniphila* could normalize the fecal targeted bacteria composition, improve the intestinal permeability, modulate inflammatory responses, and subsequently prevent liver injury in the HFD/CCl4 mice model. Following the improvement of intestinal and liver histopathology, HFD/CCl4-induced kidney damage and adipose tissue inflammation were also ameliorated by different *A. muciniphila* treatments.

In conclusion, the results of the present study indicated that oral administration of live and pasteurized *A. muciniphila* and its EVs could normalize the gut microbiota composition, improve the intestinal permeability, modulate inflammatory responses, and subsequently prevent liver injury in HFD/CCl4-administered mice. Following the improvement of intestinal and liver histopathology, HFD/CCl4-induced kidney damage and adipose tissue inflammation were also ameliorated by different *A. muciniphila* treatments (Fig. 7).

Despite these promising findings and their potential clinical implications, we acknowledge several limitations in this study. The main limitation of this study was related to specific differences in the microbiota composition and immune system of mice and humans; therefore, results should be generalized with caution. Also, we did not determine the content of EVs isolated from *A. muciniphila*; therefore, we could not fully explain the beneficial mechanism of these bacterial derivatives in liver function and intestinal hemostasis. Further studies are needed to approve the positive effects of *A. muciniphila* and its derivatives on the prevention of liver injury in humans and explore their potential risks and adverse effects. In addition, the experiments on germ-free mice are also recommended to confirm the results observed.

## MATERIALS AND METHODS

### Independent microarray data sets meta-analysis of liver fibrosis.

The seven publicly available microarray data sets were downloaded from the Gene Expression Omnibus (GEO) database at the National Center for Biotechnology Information (Table 1). Our inclusion criteria to obtain relevant data sets were as follows: (a) studies with CCl4-induced liver injury experiments, (b) mouse liver samples for the experimental setups, (c) a number of samples for each group, HC and CCl4 groups, higher than one, and (d) studies which survey the expression of all selected genes (Fig. S1). The following analyses were performed utilizing the R (version 3.6.1) statistical computing environment (82). The data in each data set were log_2_-transformed and quantile normalized, and then the probe IDs were converted into gene symbols regarding the specific annotation file of respective data sets. We eliminated the duplicity of gene symbols resulting from the multiple probe set identifiers by utilizing the average of respective entries. The meta-analyses of interest genes were conducted using the ComBat function of the SVA package, and subsequently, the limma package (version 3.40.6) was used to assess the expression profiling in case and control samples (83, 84). The adjusted *P* value of <0.05 was considered the criterion for significance.

**TABLE 1:** Characteristics of individual studies included in the meta-analysis

Study	GEO accession	GSM codes		Sample	Platform	Reference
		Fibrosis	Controls			
1	GSE21984	GSM546674	GSM546670	Liver tissue	GPL6885, Illumina MouseRef-8 v2.0 expression beadchip	24
		GSM546675	GSM546671			
2	GSE32210	GSM1047592	GSM1047598	Liver tissue	GPL4134, Agilent-014868 whole mouse genome microarray 4 × 44,000 G4122F	25
		GSM1047593	GSM1047602			
3	GSE55747	GSM1342771	GSM1342760	Liver tissue	GPL6885, Illumina MouseRef-8 v2.0 expression beadchip	26
		GSM1342772	GSM1342762			
		GSM1342773	GSM1342763			
4	GSE73985	GSM1907113	GSM1907115	Liver tissue	GPL20939, Agilent-046161 mouse LncRNA v1 8 × 60,000	27
		GSM1907114	GSM1907116			
			GSM1907117			
5	GSE80601	GSM2131445	GSM2131450	Liver tissue	GPL6096, [MoEx-1_0-st] Affymetrix mouse exon 1.0 ST array	28
		GSM2131446	GSM2131451			
		GSM2131447	GSM2131452			
		GSM2131448	GSM2131453			
		GSM2131449	GSM2131454			
6	GSE137635	GSM4083575	GSM4083573	Liver tissue	GPL11180, [HT_MG-430_PM] Affymetrix HT MG-430 PM array plate	29
		GSM4083576	GSM4083574			
7	GSE141821	GSM4213705	GSM4213687	Liver tissue	GPL18233, [MoGene-2_0-st] Affymetrix mouse gene 2.0 ST array	30
		GSM4213706	GSM4213688			
		GSM4213707	GSM4213689			
		GSM4213708	GSM4213690			
		GSM4213709	GSM4213691			
		GSM4213710	GSM4213692			
		GSM4213711	GSM4213693			
		GSM4213712	GSM4213694			
		GSM4213713				

### *A. muciniphila* growth condition.

Akkermansia muciniphila MucT (ATCC BAA-835) was obtained from the DSMZ institute (German collection of microorganisms and cell cultures). The bacterium was cultured anaerobically in a basal mucin-based medium at 37°C for 3 to 7 days (85). After that, the bacterium was inoculated into brain heart infusion (BHI) broth (Quelab, Canada) supplemented with 0.5% mucin (Sigma-Aldrich, St. Louis, MO, USA) with gentle shaking (150 rpm) under the aforementioned conditions for 48 h until an optical density at 600 nm (OD_600_) of 1 was reached. Bacterial pellets were harvested by centrifugation (11,000 × *g* for 20 min) and washed twice with sterile anaerobic PBS for cell culture treatment and oral administration in mice.

### Preparation of pasteurized *A. muciniphila*.

To prepare pasteurized bacteria, we harvested the fresh *A. muciniphila* at an OD_600_ of 1. Then, the bacterial pellets were washed twice as mentioned above, and after that, the bacterial suspension was heated at 70°C for 30 min, as described previously (13). For viability testing of the pasteurized *A. muciniphila*, the suspension was inoculated on a mucin-based medium and then incubated for at least 1 week at 37°C in the anaerobic atmosphere. The pasteurized bacteria were aliquot and stored at −80°C until used.

### EV extraction.

The supernatant obtained from *A. muciniphila* culture was filtered through a 0.22-μm filter (Sigma-Aldrich) for EVs isolation. EVs were extracted with ultracentrifuge (200,000 × *g* for 2 h at 4°C) as described previously (86). The pellets containing the EVs were resuspended in sterile PBS and stored at −80°C. Scanning electron microscopy (SEM) was used to identify the EVs morphology, and SDS-PAGE was performed to evaluate the protein content of EVs.

### Cell culture.

The LX-2 cell line, a spontaneously immortalized human HSC line, was kindly gifted from Scott L. Friedman (Mount Sinai School of Medicine, New York, NY). LX-2 cells were maintained in complete Dulbecco’s modified Eagle’s medium supplemented with 2 mM l-glutamine, 100 U/ml of penicillin, 100 μg/ml of streptomycin, and 2% heat-inactivated fetal bovine serum (Gibco-Invitrogen, Carlsbad, CA). Cells were seeded in 24-well plates and were incubated in a 5% CO_2_ humidified atmosphere at 37°C.

### LX-2 activation and treatment.

The culture medium of the LX-2 cell monolayers of 80 to 90% confluence was replaced with fresh serum-free medium overnight. For LX-2 activation, the lipopolysaccharides (LPS) from Escherichia coli 0111:B4 (Sigma-Aldrich) were used at a concentration of 0.01 μg/ml for 6 h (15). Afterwards, unstimulated LX-2 and LPS-activated cells were inoculated with live (Am) and pasteurized (Pam) *A. muciniphila* at different multiplicities of infection (MOIs 1, 10, and 100) and with EVs of various concentrations (1, 10, 50 μg/ml) for 24 h in the CO_2_ incubator at 37°C. The LPS-activated LX-2 cells and untreated cells served as the control groups. Each experiment was performed in duplicate and repeated at least three times.

### Cell viability assay.

MTT [3-(4,5-dimethyl-2-thiazolyl)-2,5-diphenyl-2H-tetrazolium bromide] assay was carried out using the cell proliferation kit I (Sigma-Aldrich) according to the manufacturer’s instructions. Briefly, LX-2 cells were seeded in 96-well plates and treated separately with the aforementioned MOIs and concentrations of bacteria and EVs for 24 h. The percentage of cell viability was calculated using the following formula: cell viability (%) = (*x* × 100%)/*y*, where “*x*” is the absorbance of treated cells and “*y*” the absorbance of untreated cells.

### Total RNA extraction and qRT-PCR analysis.

Total RNA was extracted from LX-2 cells using RNeasy plus minikit (Qiagen, Germany) following the supplier’s protocol. The quantity and quality of RNA were verified via agarose gel electrophoresis and NanoDrop spectrophotometer (ND-1000, Thermo Scientific, USA). The purified RNA was reverse-transcribed to cDNA using the BioFACT RT-kit (BIOFACT, South Korea) according to the manufacturer’s protocol. Quantitative real-time PCR (qRT-PCR) amplification was performed on a Rotor-Gene Q (Qiagen, Germany) real-time PCR system by using BioFACT 2× real-time PCR master mix (BIOFACT, South Korea), and GAPDH (glyceraldehyde-3-phosphate dehydrogenase) served as the internal control. The primers used in this study are presented in Table S1.

### Animal experiments.

Male wild-type C57BL/6 mice were obtained at the age of 7 to 8 weeks from Pasteur Institute of Iran (Tehran Iran) and housed five mice per cage for the acclimatization period. Adaptation was performed in equal conditions (12 h light, 22 to 23°C, and 40% humidity) with free access to a standard normal diet (ND) (Standard Rodent Diet A03; SAFE, Augy, France) and autoclaved water during acclimation and throughout the study. All mice were individually housed in autoclaved cages (*n *= 25) and sterile hardwood chip bedding during the experiment. This study followed the institutional guidelines regarding the care and use of laboratory animals, and all animal experiments were designed in order to minimize mice suffering. The study protocol was approved by the Animal Experiment Committee Pasteur Institute of Iran (IR.PII.REC.1399.029).

### HFD/CCl4 liver injury model and treatments.

CCl4 accompanied by a high-fat diet (260 HF 60% energy from butter, safe diet, France) was used for induction of liver injury in this study (87). The HFD consisted of butter MGLA, casein, maltodextrin, sucrose, premixture of minerals PM AIN 93M/G 3.5%, soybean oil, premixture of vitamins PV AIN 93M/G 1%, sodium bicarbonate, potassium citrate, dl-methionine, choline bitartrate, and butylhydroquinone. The HFD animals were intraperitoneally injected with 2 ml/kg body weight of 10% CCl4 solution in olive oil (Sigma-Aldrich, St. Louis, MO, USA) twice a week for 4 weeks (26). Mice were divided randomly into five groups (*n *= 5); healthy control animals received the ND only without any intervention, and Am (10^9^ CFU/200 μl live *A. muciniphila*), Pam (10^9^ CFU/200 μl pasteurized *A. muciniphila*), EVs (50 μg protein/200 μl), and vehicle (200 μl sterile PBS) were given by daily oral gavage for 4 weeks (Fig. 2A). Mice were given measured amounts of food, and food intake during the experiment was measured by weighing the food during weekly cage changes and at the end of experiment for each animal. Body weight was measured at the start of the experiment, during weekly cage changes, and at the end time point. Blood specimens were taken at the time of sacrifice from isoflurane-anesthetized mice by retrobulbar puncture without prior food fasting. Serum samples were obtained and stored at −80°C until biochemical analysis. At the end of the treatments, the mice were sacrificed by cervical dislocation, and liver, colon, adipose, and kidney tissues were snap-frozen with liquid nitrogen and stored at −80°C. The liver and colon tissues were further used for histopathological analysis.

### Histopathological analysis.

Liver and colon specimens were immediately fixed in 10% neutral buffered formalin after collection. Paraffin-embedded tissues were stained with hematoxylin and eosin (H&E) and Masson’s trichrome for liver and H&E in colon tissue. Microscopic observation of the histological slides was performed using a light microscope (88). Stained sections were evaluated by an expert pathologist, blind to study groups.

### Serum biochemical analysis.

Fasting blood glucose (Glu), total cholesterol, triglyceride (TG), low-density lipoprotein (LDL), high-density lipoprotein (HDL), very-low-density lipoprotein (VLDL), alanine transaminase (ALT), and aspartate transaminase levels were measured using a commercial kit (Bioclin-Quibasa, Belo Horizonte, MG, Brazil) in the serum samples to evaluate the liver function.

### Measurement of serum cytokines.

The level of serum IL-6, IL-10, and TNF-α was determined by using ZellBio GmbH enzyme-linked immunosorbent assay (ELISA) kit (Germany) according to the manufacturer’s instructions and analyzed with Bio-Plex Manager 6.1 software (Bio-Rad, USA). The cytokine assays were performed in duplicate.

### Western blotting.

Total protein was prepared from liver tissues using radioimmunoprecipitation assay (RIPA) lysis buffer. Cell lysates were centrifuged at 12,000 rpm for 20 min, and protein concentrations were measured using the bicinchoninic acid (BCA) protein assay (DNAbiotech, Iran). Proteins were separated by sodium dodecyl sulfate-polyacrylamide gel electrophoresis (SDS-PAGE) and then transferred to polyvinylidene difluoride (PVDF) membrane. The membrane was blocked using 3% skim milk powder at 4°C overnight and then hybridized with the primary antibodies targeting the proteins, including GADPH and α-SMA (R&D, USA, catalog number MAB5718-SP and MA5-15871, respectively). For the appearance of the protein bands, they were incubated with secondary antibodies horseradish peroxidase (HRP) conjugate goat anti-mouse IgG1 (Invitrogen, USA, catalog number A10551) for 1 h at room temperature. The blotting bands were visualized using the ECL system (Merck, Germany).

### Tissue RNA extraction and qRT-PCR analysis.

Frozen liver, colon, adipose, and kidney tissues were homogenized in 1 ml of TRIzol (33 BS410, Bio Basic, Canada) using a Precellys 24 homogenizer, and the total RNA was extracted according to the manufacturer’s instructions. The genomic DNA was removed using DNase I (Qiagen), cDNA was synthesized, and qRT-PCR amplification was performed as mentioned earlier. The relative expression of target genes was assessed using the comparative cycle threshold (Ct) method. The RPL-19 and HPRT served as the normalizer genes for tissue samples.

### Fecal DNA extraction and target microbiota analysis.

Fresh fecal samples were collected in sterile cups, immediately aliquoted in cryotubes, and stored at −80°C. Total bacterial DNA was extracted from 180 mg of each fecal sample using QIAamp DNA Stool minikit (Qiagen, GmbH, Germany) according to the manufacturer’s protocol. The DNA purity and concentration were determined by Nanodrop spectrophotometer (Thermo Scientific NanoDrop, USA). The extracted DNAs were stored at −20°C until further microbiota analysis.

Quantitative real-time PCR was performed in triplicate using Rotor-Gene Q (Qiagen, Germany) and SYBR green master mix (BIOFACT, South Korea) using specific primer pairs presented in Table S2. The amplification efficiency of each primer was determined by using the ΔCt method. The thermal cycling conditions were as follows: an initial DNA denaturation step at 95°C for 15 min, 40 cycles of denaturation at 95°C for 20 s, primer annealing at 56°C for 30 s, and extension at 72°C for 20 s. The average Ct value obtained from each reaction was transformed into percentage formula as described previously (89).

### Statistical analysis.

GraphPad Prism 8.0 (GraphPad Software Inc, CA, United States) was used to calculate changes in gene expression and cytokine production. Differences between groups were calculated using one-way analysis of variance (ANOVA) followed by Tukey’s *post hoc* test (for multiple comparisons between more than two groups) or two-way ANOVA where needed. Results are presented as the mean ± standard error of the mean of at least three experiments. The mean relative percentage of 16S rRNA genes was analyzed with the nonparametric Kruskal-Wallis test for comparison of the different groups. Spearman’s rho nonparametric correlation was performed to analyze relationships between relative abundance of microbiota, blood inflammatory and biochemical markers, and body/liver weight. These correlations were graphically visualized using Cytoscape v3.8.3 (90). A *P* value of >0.05 was considered statistically significant.
