# Supplementary material for: The Protective Effects of Live and Pasteurized Akkermansia muciniphila and Its Extracellular Vesicles against HFD/CCl4-Induced Liver Injury
Source: Microbiol Spectr. 2021 Sep 22;9(2):e00484-21. doi: 10.1128/Spectrum.00484-21 (PMC8557882; doi:10.1128/Spectrum.00484-21)
Supplement: SUPPLEMENTAL FILE 1 — Supplemental material. Download SPECTRUM00484-21_Supp_1_seq17.pdf, PDF file, 0.4 MB [file spectrum00484-21_supp_1_seq17.pdf]

Table S1. Oligonucleotide primers used in real-time PCR in cell line and mice.

Target gene	Primer designation	Oligonucleotide sequence (5`-3`)	Product size (bp)
**m-TLR-2**	Tlr_2-F	TCCTGCGAACTCCTATCC	151
	Tlr_2-R	CCTGGTGACATTCCAAGAC	
**m-TLR-4**	Tlr_4-F	GCCTTTCAGGGAATTAAGCTCC	114
	Tlr_4-R	GATCAACCGATGGACGTGTAAA	
**m-TNF-α**	Tnf-F	AACAACTACTCAGAAACACAAG	130
	Tnf-R	GCAGAACTCAGGAATGGA	
**m-TGF-β**	Tgfb-F	AATTCCTGGCGTTACCTT	116
	Tgfb-R	TGTATTCCGTCTCCTTGG	
**m-α-SMA**	Sma-F	CTGCCGAGCGTGAGATTG	122
	Sma-R	AGGCAGTTCGTAGCTCTTCT	
**m-Col1a1**	Col1-F	GGGGCAAGACAGTCATCGAA	144
	Col1-R	GGGTGGAGGGAGTTTACACG	
**m-TIMP1**	Timp-R	AGTGTTTCCCTGTTTATCTATCC	105
	Timp-R	AAGTGACGGCTCTGGTAG	
**m-PDGF**	Pdgf-F	CACCACCGCAGTGTCAAG	116
	Pdgf-R	AGGTTGGAGGTCGCACAT	
**m-RPL-19**	Rpl-F	TCAGCCACAACATTCTCA	138
	Rpl-R	GCACCTCCAACAGTAAGT	
**m-HPRT**	Hprt-F	TCAGTCAACGGGGGACATAAA	142
	Hprt-R	GGGGCTGTACTGCTTAACCAG	
**TLR-2**	Tlr_2-F	TTATCCAGCACACGAATACACAG	160
	Tlr_2-R	AGGCATCTGGTAGAGTCATCAA	
**TLR-4**	Tlr_4-F	AGACCTGTCCCTGAACCCTAT	147
	Tlr_4-R	CGATGGACTTCTAAACCAGCCA	
**TGF-β**	Tgfb-F	GCACAACTCCGGTGACATCAA	86
	Tgf-R	CAATTCCTGGCGATACCTCAG	
**α-SMA**	Sma-F	ATGATGCTGTTGTAGGTG	142
	Sma -R	TTGAGAAGAGTTACGAGTTG	
**Col1a1**	Col1-F	TTACACAAGGAACAGAACA	91
	Col1-R	CGACAAAGCAGAAACATC	

**TIMP1**	Timp-F	CTATCAGCCACAGCAACAACAGG	82
	Timp-R	GCCCAGAGAGACACCAGAGAAC	
**GAPDH**	GAPDH-F	TGGAAGATGGTGATGGGATT	211
	GAPDH-R	TCAACGGATTTGGTCGTATTG	

Table S2. Oligonucleotide primers used for the gut microbiota analysis.

Target gene	Primer designation	Oligonucleotide sequence (5`-3`)	Product size (bp)
**Universal**	Uni-F<br>Uni-R	ACTCCTACGGGAGGCAGCAGT<br>ATTACCGCGGCTGCTGGC	[1]
**Firmicute**	Firm-F<br>Firm-R	GGAG**Y**ATGTGGTTTAATTCGAAGCA<br>AGCTGACGACAACCATGCAC	[2]
**Bacteroidetes**	Bactero-F<br>Bactero-R	GTTTAATTCGATGATACGCG<br>TTAAGCCGACACCTCACG	[2]
**Fusobacteria**	Fuso-F<br>Fuso-R	GATCCAGCAATTCTGTGTG<br>CGAATTTCACCTCTACACTTG	[3]
**Actinobacteria**	Actino-F<br>Actino-R	GCGACCTATCAGCTTGTT<br>CCGCCTACGAGCTCTTTACGC	[3]
**Clostridia**	Ccl-F<br>Ccl-R	AAAGGAAGATTAATACCGCATA<br>TTCTTCCTAATCTCTACGCA	[4]
**γ-Proteobacteria**	Gamma-F<br>Gamma -R	CATGCCGCGTGTGTGAA<br>ACTCCCCAGGCGGTC**D**ACTTA	[5]
**α-Proteobacteria**	Alpha-F<br>Alpha-R	GGTAAGGTTCTGCGCGTT<br>GGTAAGGTTCTGCGCGTT	[5]
**ε-Proteobacteria**	Epsilon-R<br>Epsilon-R	TGGTGTAGGGGTAAAATCCG<br>AGGTAAGGTTCTTCG**Y**GTATC	[3]
Enterobacteriaceae	Enterob-F<br>Enterob-R	CGTCGCAAG**MM**CAAAGAG<br>TTACCGCGGCTGCTGGCAC	[3]
Rumminococcaceae	Rum-F<br>Rum-R	GGCGGC**Y**T**R**CTGGGCTTT<br>CCAGGTGGAT**W**ACTTATTGTGTTAA	[6]
Peptostreptococcus	Pepto-F<br>Pepto-R	ATAGGAGGAAGCCCTGGCTAAA<br>CTCCACGCTTTGACACCTGA	This study
Prevotellaceae	Prevo-F<br>Prevo-R	CACCAAGGCGACGATCA<br>GGATAACGCCTGGACCT	[7]
Methanobrevibacter spp.	Methano-F<br>Methano-R	CGATGCGGACTTGGTGTTG<br>TGTCGCCTCTGGTGAGATGTC	[8]

A. muciniphila	Am-F	CAGCACGTGAAGGTGGGGAC	[9]
	Am-R	CCTTGCGGTTGGCTTCAGAT	
Alistipes spp.	Alis-F	TTAGAGATGGGCATGCGTTGT	[10]
	Alis-R	TGAATCCTCCGTATT	
Veillonella spp.	Veillo-F	ACAACCTGCCCTTCAGA	[11]
	Veillo-R	CGTCCCGATTAACAGAGCTT	
Enterococcus spp.	Enteroc-F	CCCTTATTGTTAGTTGCCATCATT	[12]
	Enteroc-R	ACTCGTTCTACTTCCCATTGT	
F. prausnitzii	Fp-F	GATGGCCTCGCGTCCGATTAG	[13]
	Fp-R	CCGAAGACCTTCTTCCTCC	
E. coli	GAPDH-F	TGGAAGATGGTGATGGGATT	[14]
	GAPDH-R	TCAACGGATTTGGTCGTATTG	
Bifidobacterium spp.	Bifido-F	GGGATGCTGGTGTGGAAGAG	[12]
	Bifido-R	TGCTCGCGTCCACTATCCAG	
Lactobacillus spp.	Lacto-F	TGGATGCCTTGGCACTAG	[12]
	Lacto-R	AAATCTCCGGATCAAAGCTTAC	
Roseburia spp.	Rose-F	TACTGCATTGGAAACTGTCG	[7]
	Rose-R	CGGCACCGAAGAGCAAT	

The nucleotides in bold type represent: Y, C or T; K, G or T; M, A or C; D, A or G or T; R, A or G.

Figure S1. Flow chart for database search.

Figure S2. Gene expression of liver fibrosis markers in LX-2 cells upon treatment with different MOIs of live, pasteurized *A. muciniphila* and its EVs. Relative gene expression of fibrosis markers were markedly increased after treatment of LX-2 cells with LPS in comparison with quiescence condition. The mRNA level of an anti-fibrosis factors were decreased in LPS stimulated LX-2 cells treated with different supplementation of *A. muciniphila*. Data are shown as the mean ± SEM. *$P < 0.05$, **$P < 0.01$, ***$P < 0.001$ and by post hoc one-way ANOVA statistical analysis.

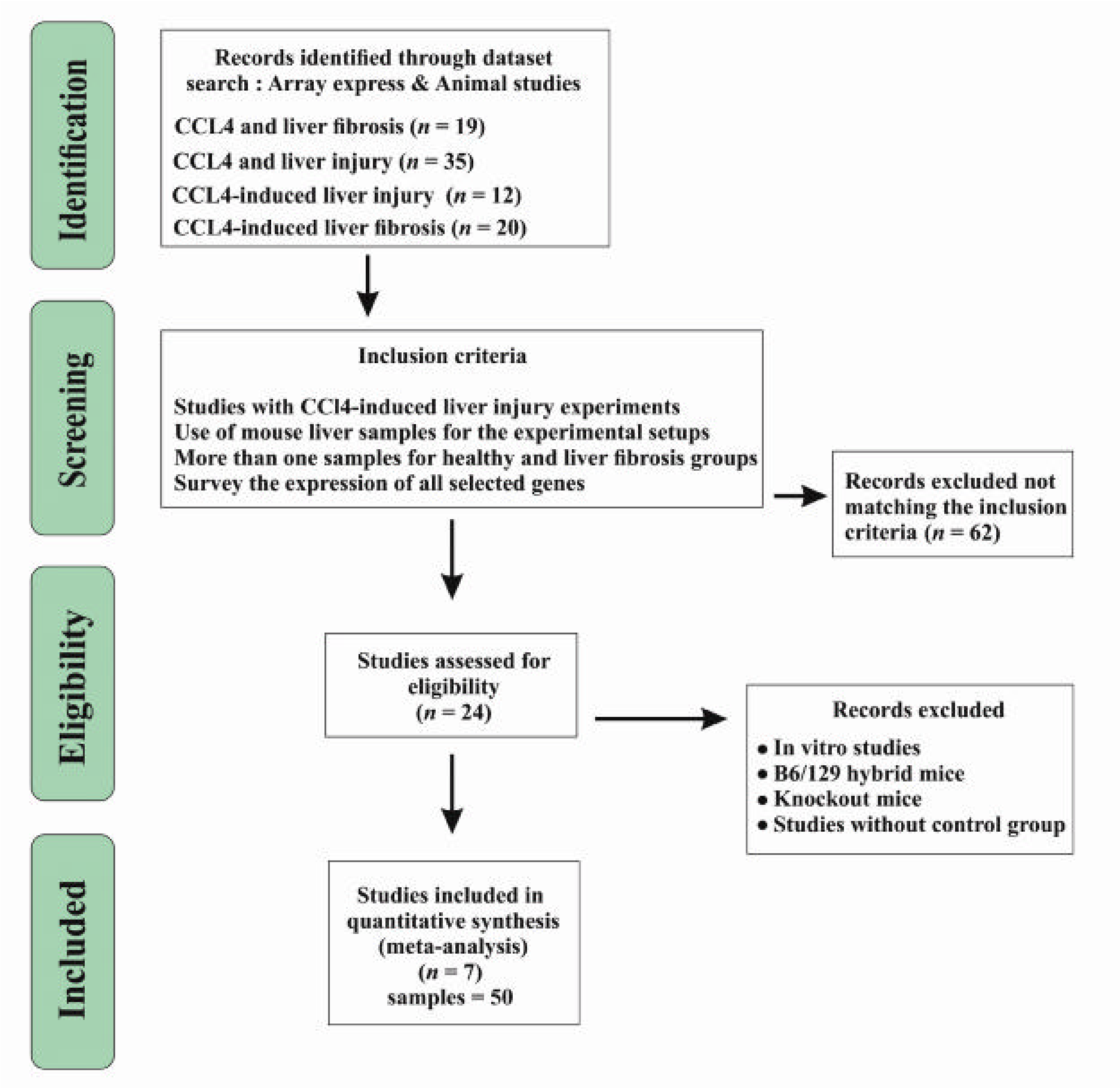

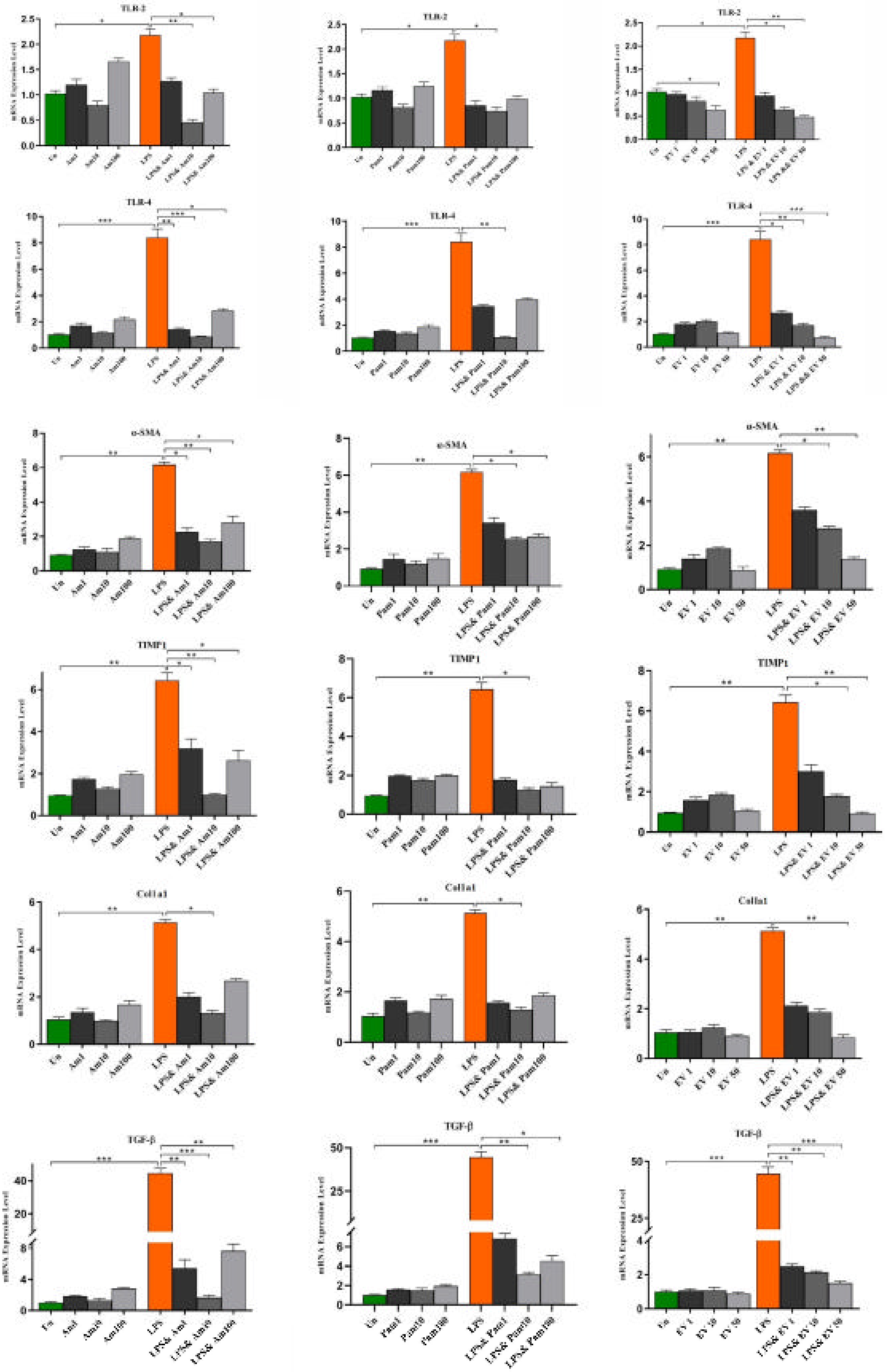
TLR-2
TLR-4
α-SMA
TIMP1
Col1a1
TGF-β
mRNA Expression Level
Un
Am1
Am10
Am100
LPS
LPS& Am1
LPS& Am10
LPS& Am100
Pam1
Pam10
Pam100
LPS& Pam1
LPS& Pam10
LPS& Pam100
EV 1
EV 10
EV 50
LPS& EV 1
LPS& EV 10
LPS& EV 50

Reference

1 Moraes, J. G. d., Motta, M. E. F. d. A., Beltrão, M. F. d. S., Salviano, T. L. & Silva, G. A. P. d. Fecal microbiota and diet of children with chronic constipation. *International journal of pediatrics*. 2016; **2016**, p. 6787269.

2 Yang, Y.-W. *et al.* Use of 16S rRNA gene-targeted group-specific primers for real-time PCR analysis of predominant bacteria in mouse feces. *Applied and environmental microbiology*. 2015; **81**, p.6749-6756.

3 Hermann-Bank, M. L., Skovgaard, K., Stockmarr, A., Larsen, N. & Mølbak, L. The Gut Microbiotassay: a high-throughput qPCR approach combinable with next generation sequencing to study gut microbial diversity. *BMC genomics*. 2013; **14**, p.788.

4 Matsuki, T. *et al.* Development of 16S rRNA-gene-targeted group-specific primers for the detection and identification of predominant bacteria in human feces. *Applied and environmental microbiology*. 2002; **68**, p.5445-5451.

5 De Gregoris, T. B., Aldred, N., Clare, A. S. & Burgess, J. G. Improvement of phylum-and class-specific primers for real-time PCR quantification of bacterial taxa. *Journal of microbiological methods*. 2011; **86**, p.351-356.

6 Omar, J. M., Chan, Y.-M., Jones, M. L., Prakash, S. & Jones, P. J. Lactobacillus fermentum and Lactobacillus amylovorus as probiotics alter body adiposity and gut microflora in healthy persons. *Journal of functional foods*. 2013; **5**, p.116-123.

7 Larsen, N. *et al.* Gut microbiota in human adults with type 2 diabetes differs from non-diabetic adults. *PloS one*. 2010; **5**, e9085.

8 Verma, R., Verma, A. K., Ahuja, V. & Paul, J. Real-time analysis of mucosal flora in patients with inflammatory bowel disease in India. *Journal of clinical microbiology*. 2010; **48**, p.4279-4282.

9 Schneeberger, M. *et al.* Akkermansia muciniphila inversely correlates with the onset of inflammation, altered adipose tissue metabolism and metabolic disorders during obesity in mice. *Scientific reports*. 2015; **5**, p.16643.

10 Vigsnæs, L. K., Brynskov, J., Steenholdt, C., Wilcks, A. & Licht, T. R. Gram-negative bacteria account for main differences between faecal microbiota from patients with ulcerative colitis and healthy controls. *Beneficial microbes*. 2012; **3**, p.287-297.

11 Rinttilä, T., Kassinen, A., Malinen, E., Krogius, L. & Palva, A. Development of an extensive set of 16S rDNA-targeted primers for quantification of pathogenic and indigenous bacteria in faecal samples by real-time PCR. *Journal of applied microbiology*. 2004; **97**, p.1166-1177.

12 Wang, I.-K. *et al.* Real-time PCR analysis of the intestinal microbiotas in peritoneal dialysis patients. *Applied and environmental microbiology*. 2012; **78**, p.1107-1112.

13 Fitzgerald, C. B. *et al.* Comparative analysis of Faecalibacterium prausnitzii genomes shows a high level of genome plasticity and warrants separation into new species-level taxa. *BMC genomics.* 2018; **19**, p.931.

14 Bartosch, S., Fite, A., Macfarlane, G. T. & McMurdo, M. E. Characterization of bacterial communities in feces from healthy elderly volunteers and hospitalized elderly patients by using real-time PCR and effects of antibiotic treatment on the fecal microbiota. *Applied and environmental microbiology*. 2004; **70**, p.3575-3581.
